# *Wolbachia* Host Shifts and Widespread Occurrence of Reproductive Manipulation Loci in European Butterflies

**DOI:** 10.1111/mec.70125

**Published:** 2025-10-08

**Authors:** Eric Toro‐Delgado, Dominik R. Laetsch, Alex Hayward, Gerard Talavera, Konrad Lohse, Roger Vila

**Affiliations:** ^1^ Institut de Biologia Evolutiva (CSIC – Universitat Pompeu Fabra) Barcelona Spain; ^2^ Departament de Biologia Evolutiva, Ecologia i Ciències Ambientals Universitat de Barcelona Barcelona Spain; ^3^ Institute of Ecology and Evolution University of Edinburgh Edinburgh UK; ^4^ Centre for Ecology and Conservation University of Exeter Cornwall UK; ^5^ Institut Botànic de Barcelona (IBB), CSIC‐CMCNB Barcelona Spain

**Keywords:** co‐cladogenesis, Lepidoptera, reproductive manipulation, sister species, symbiosis, *Wolbachia*

## Abstract

*Wolbachia* is the most frequent bacterial endosymbiont of arthropods and nematodes. Although it is mostly vertically transmitted, from parent to offspring through the egg cytoplasm, horizontal transfer of *Wolbachia* is thought to be common over evolutionary timescales. However, the relative frequency of each transmission mechanism has not been studied systematically in closely related species. Additionally, while *Wolbachia* is generally regarded as a reproductive manipulator, it is unclear how frequently the symbiont induces such effects. In this study, we investigated the presence, phenotypes and phylogenetic relationships among *Wolbachia* strains in whole genome sequence data for 18 European butterfly sister‐species pairs. We find that sister‐species share *Wolbachia* strains more often than random species pairs and that the probability of strain sharing is higher for younger pairs of host species, especially those with greater range overlap. We also find that split times between *Wolbachia* strains that infect the same sister‐species pair generally pre‐date host divergence, ruling out co‐divergence in favour of horizontal transfer. However, some strains are younger than the mitochondrial split times of their hosts, so introgressive transfer cannot be ruled out in some cases. In addition, all newly assembled *Wolbachia* genomes contained putative homologues of genes associated with cytoplasmic incompatibility and male killing. This supports the potential for reproductive manipulation in *Wolbachia* strains infecting European butterflies, which until now was only inferred from mitochondrial diversity patterns. Our results show that horizontal and introgressive transfer of *Wolbachia* are frequent even between recently speciated host taxa, suggesting the symbiont's turnover rate is higher than had been inferred previously from surveys of distantly related hosts.

## 1 Introduction

*Wolbachia* is a genus of Alphaproteobacteria that lives as intracellular endosymbiont of arthropods and nematodes, and is primarily transmitted vertically through the egg cytoplasm. Although the taxonomy of the genus is unclear, *Wolbachia* is classified into distinct genetic clades or ‘supergroups’ (Lo et al. 2007); whether such supergroups constitute distinct species is a matter of debate (Ellegaard et al. 2013). In arthropods, where the estimated incidence (fraction of species infected) is ~52% (Weinert et al. 2015), *Wolbachia* mainly acts as a reproductive parasite that manipulates the host to increase its spread by several mechanisms. These include male killing (MK), in which only female offspring survive to adulthood (Sakamoto et al. 2007); feminization, in which male offspring develop as females; induced parthenogenesis, in which infected females produce female offspring without mating; and cytoplasmic incompatibility (CI), in which crosses between uninfected females and infected males result in fewer viable offspring (Werren et al. 2008). However, *Wolbachia* has also been found to have positive fitness effects in some cases, such as pathogen protection (Hedges et al. 2008; Osborne et al. 2009; Tong et al. 2021; Ye et al. 2013) or nutrient provisioning (Gerth and Bleidorn 2016; Nikoh et al. 2014).

Given its effects on host reproduction, *Wolbachia* has been hypothesised to influence host speciation. The simplest scenario in which *Wolbachia* can generate reproductive isolation (RI) is bidirectional CI, where different, mutually incompatible strains become fixed in two populations, resulting in nonviable crosses. In theory, if strong bidirectional CI is maintained for long enough, host populations will develop other reproductive incompatibilities. Thus, *Wolbachia* could facilitate host speciation, even when only present during the early stages of the process (Bruzzese et al. 2022). However, loss of *Wolbachia* due to imperfect maternal transmission (Adekunle et al. 2019; Reuter et al. 2005) or replacement by another strain (Kriesner et al. 2013) could break down *Wolbachia*‐mediated RI. Host population structure, in particular migration rates across subpopulations differing in infection status, also affects the chances of RI arising among these subpopulations (Engelstädter and Telschow 2009). Therefore, the role of *Wolbachia* in host speciation is still largely debated.

Determining the tempo and mode of *Wolbachia* acquisition and loss (i.e., the turnover rate) is essential to understand the potential of *Wolbachia* to co‐evolve with its hosts and its effects on host speciation. Bailly‐Bechet et al. (2017) used a co‐phylogenetic analysis of *Wolbachia* and DNA barcoding data of hosts from the Society Archipelago (French Polynesia) to estimate rates of *Wolbachia* loss and gain of 0.14 and 0.11 per million years, respectively. However, their study is based on taxonomically disparate host samples from a small and remote archipelago, limiting its applicability to continental areas. In addition, they only analyzed mitochondrial data, which cannot discriminate between all transmission mechanisms. Therefore, more focused comparative studies that examine patterns of *Wolbachia* strain sharing between closely related host species are necessary to better understand *Wolbachia*–host evolution.

Alternative scenarios of *Wolbachia* evolution are distinguishable by their contrasting co‐phylogenetic patterns: if *Wolbachia* transmission was entirely vertical, with high persistence, we would expect topological congruence between host and symbiont phylogenies (i.e., *Wolbachia* and host phylogenies mirror each other) and split times, as *Wolbachia* lineages would co‐diverge with the corresponding host lineages (i.e., co‐cladogenesis). Alternatively, if *Wolbachia* were frequently acquired through horizontal symbiont transfer (hereafter, horizontal transfer), with high turnover, we would expect topologically incongruent host and symbiont phylogenies. A third scenario is introgressive transfer, that is, the transfer of *Wolbachia* strains between host species by hybridisation. In this case, as the transfer is still maternal, the mitochondrial genome of the introgressing species hitchhikes with *Wolbachia*, leading to incongruent host nuclear and symbiont phylogenies, but congruence between *Wolbachia* and host mitochondrial phylogenies. If the event is recent enough, both host species share mitochondrial haplotypes. Both horizontal and introgressive transfer result in a host shift, that is, the movement of a given *Wolbachia* lineage into a new host that the symbiont's immediate ancestors did not infect (de Vienne et al. 2013).

Past studies investigating *Wolbachia* co‐phylogenetic patterns in arthropods found no or very limited evidence for co‐cladogenesis, suggesting that introgressive and horizontal transfer are frequent and that the persistence of *Wolbachia* strains on their hosts is low (Ahmed et al. 2016; Raychoudhury et al. 2009; Shoemaker et al. 2002; Sontowski et al. 2015). For example, Turelli et al. (2018) found that a *Wolbachia* strain spread across eight different *Drosophila* species in less than 30,000 years, with the comparison of symbiont and mitochondrial phylogenies and split times supporting both introgressive and horizontal transfer. Similarly, Schuler et al. (2013) investigated introduced populations of *Rhagoletis cingulata* in Europe, finding that a *Wolbachia* strain from the European native species *Rhagoletis cerasi* managed to spread into *R. capitata* since its introduction at the end of the 20th century, despite the two species being distantly related (~13% mitochondrial divergence). Ecological interactions provide a mechanism for *Wolbachia* horizontal transfer, for example through shared food resources (e.g., leaf beetles and their host plants, Cardoso and Gómez‐Zurita 2020; or Diptera feeding on the same fungi, Stahlhut et al. 2010), parasitoids (e.g., among hosts, Ahmed, Li, et al. 2015; or from host to parasitoid, Heath et al. 1999) or mutualists (e.g., ants tending scale‐insects, Sanaei et al. 2022). On the other hand, long‐term persistence of a given *Wolbachia* infection has also been found in some cases. For example, Gerth et al. (2013) investigated *Wolbachia* transfer in bees, identifying a potential case of co‐cladogenesis among three closely related *Nomada* species dating back to 1.7 Mya.

However, many studies of *Wolbachia* transmission and turnover are based on the sequencing of a few selected genes, especially the *Wolbachia* surface protein gene (*wsp*) and the Multilocus Sequence Typing (MLST) system (Baldo et al. 2006). These approaches, in particular MLST, have been found to have limited reliability for phylogenetic inference and strain differentiation (Bleidorn and Gerth 2018). In contrast, analyses of whole genome data can resolve very recently diverged *Wolbachia* strains. In addition, while taxonomically broad comparisons of host and *Wolbachia* phylogenies have found ample evidence for horizontal and introgressive transfer, vertical transmission (which, if always maintained, would lead to strict co‐divergence) remains the main transmission mechanism at an intraspecific level (Richardson et al. 2012; Schuler et al. 2018). Thus, investigating *Wolbachia* transmission between recently diverged host sister species pairs may inform about the timescale at which host shifts occur.

In butterflies (superfamily Papilionoidea), a large proportion of species are known to be infected with *Wolbachia*, with an estimated incidence between 65% and 95% and a mean prevalence (fraction of infected specimens in a species) between 22% and 29% (Ahmed et al. 2015). Cases of *Wolbachia*‐induced CI, MK, and feminization have been documented (Duplouy and Hornett 2018). It has also been shown that the phenotypes of a strain within a given host can change rapidly as a result of coevolution (Hornett et al. 2008), and that host individuals can be co‐infected with multiple strains that cause different reproductive manipulations (Hiroki et al. 2004). *Wolbachia* has also been found to manipulate sex chromosome inheritance in the butterfly *Eurema mandarinia* (Kageyama et al. 2017). *Wolbachia*‐induced CI is frequently invoked as an explanation for patterns of mitonuclear discordance in butterflies. For example, Gaunet et al. (2019) proposed a *Wolbachia*‐mediated genetic sweep to explain the observed mitonuclear discordance in *Iphiclides*, while Kodandaramaiah et al. (2013) found two different mitochondrial clades associated with distinct *Wolbachia* strains in *Coenonympha tullia* . However, the effects of most strains infecting butterflies have not been directly characterised, as this requires crossing experiments. The recent characterisation of the candidate loci involved in CI (Beckmann et al. 2017; Le Page et al. 2017) and MK (Katsuma et al. 2022; Perlmutter et al. 2019) allows for indirect inference of the potential effects of a given strain.

While there is some degree of phylogenetic conservatism between supergroup B *Wolbachia* and Lepidoptera (Vancaester and Blaxter 2023), most studies of *Wolbachia* diversity within butterflies found no or very little topological congruence between host and symbiont phylogenies. This suggests that introgressive and horizontal transfer (possibly mediated by parasitoids; Batista et al. 2010) of different B *Wolbachia* strains is frequent within Lepidoptera (Duplouy et al. 2020; Zhao et al. 2021). On the other hand, case studies focusing on closely related butterfly hosts have revealed some evidence for co‐cladogenesis (mixed with introgressive and horizontal transfer) in the genera *Erebia* (Lucek et al. 2021) and *Plebejus* (as *Lycaeides*; Shastry et al. 2022).

Here we conduct a systematic study of *Wolbachia* turnover in butterflies and investigate the presence, putative phenotypes and phylogenetic relationships of *Wolbachia* strains in 18 butterfly sister species pairs, covering 18 genera across all five major families in Europe, to ask the following questions:

1. Do butterfly sister‐species show evidence of *Wolbachia* co‐cladogenesis, or are introgressive and horizontal transfers pervasive at this scale?
2. How frequent are CI‐ and MK‐inducing loci in *Wolbachia* strains infecting butterflies?

Our study represents a taxonomically comprehensive, WGS‐based *Wolbachia* screen in the European butterfly fauna. We show that introgressive and horizontal transfer are frequent even in recently diverged sister species, suggesting these phenomena are relevant at shorter timescales than may be expected from previous studies. In addition, we also confirm the presence of CI and MK loci among *Wolbachia* strains in European butterflies, which until now have only been inferred from patterns of mitochondrial diversity.

## 2 Materials and Methods

### 2.1 Sampling and Data Collection

Butterflies were collected between 2006 and 2019 in multiple European countries. Samples were either dried and stored in absolute ethanol at −20°C or flash frozen at −80°C in a dry shipper and stored at −80°C. Species identifications of morphologically similar species were confirmed using the barcoding region of the cytochrome *c* oxidase subunit I gene, in cases where this marker is considered to be diagnostic (see Ebdon et al. 2021 for details). We also conducted principal component analysis (PCA) of the genetic variation within each genus to confirm species identifications (Figure S1). Sister‐species pairs were selected based on a complete phylogeny of European butterflies (Wiemers et al. 2020) and phylogenies of specific genera (Peña et al. 2015); a total of 18 pairs were selected, which constitutes a representative fraction of recent butterfly speciation events across all European butterfly families (except Riodinidae, with only one species in Europe). These species are very heterogeneous, with a wide range of sizes (12–46 mm of forewing length), host plant families (e.g., Poaceae, Fabaceae, Rosaceae) and habitats (from lowland to montane specialists), and the pairs range in split times from 0.92 (*Colias*) to 8.5 Mya (*Satyrus*). In eight cases, specimens of a third species in the same genus were also obtained and sequenced, resulting in a total of 44 species. These additional species were included in analyses beyond the pairwise comparisons.

Two types of genomic data were generated. For a subset of 26 specimens (comprising at least one per genus), we generated both PacBio contiguous long‐read (CLR) data (expected coverage 50×) and Illumina paired‐end short‐read data (expected coverage 25×) for a single sample (the ‘genomic reference specimens’), from which *Wolbachia* genome assemblies were generated. In addition, we generated Illumina paired‐end libraries at the same expected coverage for at least six samples from throughout the range of each species (the ‘resequence specimens’). This resulted in short read data for a total of 273 specimens (Table S1).

To generate PacBio libraries for the genomic reference samples, high molecular weight (HMW) DNA was extracted from flash frozen specimens using thorax and abdominal tissue and a salting‐out protocol described in Mackintosh et al. (2022a, 2022b). For each of the 26 genomic reference individuals, we performed a single HMW extraction, and PacBio SMRTbell libraries were prepared and sequenced on a Sequel instrument to generate CLR data.

Short‐read WGS data were generated from a mixture of either −80°C or ethanol‐preserved samples (Table S1). DNA was extracted from thoracic tissue using a Qiagen Blood and Tissue kit following the manufacturer's protocol. Extractions were treated with RNAseA (1 h at 37°C), eluted in a total volume of 80 μL, and DNA quantified using Qubit. TruSeq DNA libraries (350 bp inserts; either Nano or PCR free) were generated by Edinburgh Genomics and sequenced on a HiSeq X or NovaSeq SP instrument (150 base paired‐end reads, sample).

In addition, we also retrieved *Wolbachia* reference genomes from the NCBI RefSeq database (Sayers et al. 2022; *N* = 76; downloaded on October 7, 2022) and from the Darwin Tree of Life (DToL) project (Vancaester and Blaxter 2023; *N* = 110; downloaded on December 11, 2022). As *Wolbachia* strains found in a given insect order do not form monophyletic clades (Vancaester and Blaxter 2023), we downloaded strains from all available arthropod and nematode hosts.

### 2.2 *Wolbachia* and Mitochondrial Genome Assemblies

Illumina libraries were trimmed on both their 5′ and 3′ ends using FASTP (Chen et al. 2018), with a mean quality threshold of 20 and a window size of four bases. Afterwards, reads were mapped against the reference genome of the closest available butterfly species to check host coverage, and those with a mean coverage lower than 5× were discarded.

For each host butterfly, the nuclear genome had been previously assembled from the corresponding read libraries (Mackintosh et al. 2022a, 2022b). If the butterfly was infected, the genome assembly can already contain the *Wolbachia* genome as one of the contigs. Therefore, we used BlobTools (Laetsch and Blaxter 2017a) to assess the taxonomic identities of the contigs obtained during the assembly of the butterfly genomes. If *Wolbachia* contigs were found and BUSCO (Manni et al. 2021) completeness (based on the rickettsiales_odb10 dataset) was ≥ 90%, they were kept as reference *Wolbachia* genomes. If *Wolbachia* contigs were found but with lower BUSCO completeness, PacBio and Illumina libraries were mapped back to the assembled butterfly genome using minimap2 (Li 2018), and we extracted all reads from both libraries that did not map to any contig, or those that mapped to either *Wolbachia* contigs or to contigs of uncertain taxonomic assignment. Using these reads, a new long‐read assembly with short‐read polishing was carried out, using NextDenovo (Hu et al. 2024) and HAPO‐G (Aury and Istace 2021). If an assembly could not be obtained with this strategy, we performed long‐read assembly with Flye (Kolmogorov et al. 2020) and short‐read polishing with HAPO‐G; the minimap2 and BlobTools steps were repeated to partition the reads mapping to *Wolbachia* contigs only, and assembly with these reads was attempted. A summary of the generated assemblies can be found in Table S2.

For the mitochondrial assemblies, the Illumina paired‐end libraries were used. If a genomic reference specimen was available, we used the Illumina library of that specimen; otherwise, we took the resequenced specimen with the highest coverage. We did not use the long‐read data for assembly because de novo assembly with high‐quality short reads proved more effective than trying to recover mitogenomes from the noisy long reads. We tried several combinations of MitoHifi (Uliano‐Silva et al. 2023), NOVOplasty (Dierckxsens et al. 2017) and mitoZ (Meng et al. 2019) for assembly and annotation, and chose the best assembly based on the quality of the annotation (Table S3). In the cases where we used a DToL reference genome, we took the mitochondrial sequence from that assembly and annotated it to obtain the protein‐coding genes.

### 2.3 Phylogenetic Inference and Co‐Phylogenetic Analyses

The newly assembled *Wolbachia* genomes, together with those obtained from DToL, were annotated using Prokka (Seemann 2014), using the proteomes of all RefSeq *Wolbachia* genomes to guide the annotation. The resulting proteomes, together with the RefSeq ones, were used to infer orthogroups with Orthofinder (Emms and Kelly 2015) and KinFin (Laetsch and Blaxter 2017b), with the exceptions of GCF_918315375.1 and GCF_918308635.1, which were discarded because they were represented in less than 20% of the orthogroups and had BUSCO completeness < 40%. We recovered 250 single‐copy orthologs (SCOs) present in 95% of the genomes. These SCOs were then aligned using MAFFT (Katoh 2002), trimmed using trimAl (Capella‐Gutiérrez et al. 2009), and concatenated with FASconCAT‐G (Kück and Longo 2014) or SuperCRUNCH (Portik and Wiens 2020) to build a supermatrix that was used as input to build a maximum‐likelihood phylogeny of all *Wolbachia* genomes (*N* = 195) with IQ‐TREE (Minh et al. 2020). Substitution model parameters were estimated separately for each orthogroup, using the GTR+Γ substitution model. 1000 replicates were run for both the ultra‐fast bootstrap with hill‐climbing NNI and the SH‐aLRT. Since the resultant phylogeny was consistent with previous results with regard to supergroup classification (Vancaester and Blaxter 2023) and all our assembled genomes belonged to supergroup B, the same approach was repeated to build a phylogeny of only supergroup B *Wolbachia*, keeping three supergroup A taxa as outgroups (*N* = 88). This phylogeny was based on 493 SCOs present in at least 95% of the strains.

In addition, we built both nuclear and mitochondrial phylogenies for the butterfly hosts, using a combination of our data and publicly available genomes (Darwin Tree of Life Project Consortium 2022; Hayward et al. 2022; Lohse et al. 2023; Hayward et al. 2022; Lohse et al. 2021; Lohse et al. 2022; Lohse et al. 2022; Mackintosh et al. 2023, 2024; Mackintosh et al. 2022a, 2022b; Vila et al. 2022). For the nuclear phylogeny, we proceeded as follows: for species without a reference genome, we mapped the highest coverage Illumina library among the resequenced individuals to the closest available reference genome using minimap2. Then, with the output from BUSCO for the reference genome, we used BEDTools (Quinlan and Hall 2010) to select those regions corresponding to the BUSCO genes, extended 1000 bp upstream and downstream to allow for variation in the exact positioning of BUSCOs in the resequenced individuals. BAM files were subsetted with SAMtools (Danecek et al. 2021) to keep only the reads mapped to the selected regions and with mapping quality > 20, and variants were called from these subsetted BAM files and filtered based on quality ≥ 20 and number of reads ≥ 8 using BCFtools (Danecek et al. 2021). Afterwards, a phylogeny was built following the same approach as for *Wolbachia*, but using the set of single‐copy BUSCOs from the BUSCO analysis of each genome or consensus sequence. A total of 4186 BUSCOs present in at least 95% of the samples were used. For the mitochondrial, the same supermatrix approach as for the nuclear and *Wolbachia* phylogenies was used, by aligning, trimming and concatenating the protein sequences of the 13 mitochondrial protein‐coding genes extracted from the previously assembled mitogenomes.

Finally, we built tanglegrams and interaction matrices and measured the co‐phylogenetic signal between the butterfly and *Wolbachia* phylogenies using Random TaPas (Balbuena et al. 2020), with PACo (Hutchinson et al. 2017) as the global‐fit test to assess each tanglegram partition. We conducted two sets of analyses, one comparing the host nuclear phylogeny to the *Wolbachia* one and another comparing the mitochondrial and the symbiont ones. In both cases, Random TaPas was run 10 times to obtain a robust estimate of the average Gini coefficient.

### 2.4 Detection of *Wolbachia* Presence in Illumina Libraries

We determined the presence and number of *Wolbachia* strains in each sample by mapping the corresponding Illumina libraries to the *Wolbachia* reference genomes (both newly assembled and downloaded). Given that *Wolbachia* reference genomes vary in terms of their similarity and some can be highly similar and arguably represent the same strain, first we conducted a dereplication analysis using dRep (Olm et al. 2017) to cluster those genomes with an average nucleotide identity (ANI) higher than 99% across a minimum aligned fraction (MAF) of 90%. Then, using the set of dereplicated genomes, we conducted a competitive mapping of each Illumina library against the concatenation of the dereplicated genomes using minimap2, and evaluated the breadth and depth of coverage for each of the concatenated genomes using inStrain (Olm et al. 2021). This step revealed samples with almost no mapped reads to any genome, which were considered uninfected, and samples with substantial mapping to multiple genomes, which were considered infected (Table S4).

The infected samples were then progressively mapped to an increasing number of genomes, starting with the best‐mapping one, to infer the number of strains (Table S5). For each sample, the reference genome with the highest breadth of coverage in the initial competitive mapping was selected to map against. Then, depth and breadth of coverage were evaluated again, and the percent of ‘heterozygous’ sites over the total of covered bases in the reference genome was calculated with a custom Python script using the Pysam package (Bonfield et al. 2021; Danecek et al. 2021; Pysam‐Developers 2024). If breadth was above 90%, the sample was confirmed as infected. If the number of ‘heterozygous’ positions was higher than 10 sites per kilobase (which is equivalent to an ANI < 99%), we considered that the sample was likely coinfected with another strain. In such a case, we mapped the Illumina library again against the concatenation of the top two genomes with the highest breadth of coverage in the initial competitive mapping step, and re‐evaluated the number of ‘heterozygous’ sites for each of the reference genomes. If it was still higher than 10 sites per kilobase, the same steps were repeated again, adding the third highest breadth reference genome, and so on until the number of ‘heterozygous’ sites was lower than 10 sites per kilobase. The genomes mapped against in this last iteration were considered as representing the strains found in that sample. However, given the stringent thresholds for dereplication of the reference genome set, some representative genomes can be highly similar across the fraction that they align. This could create a situation in which two similar genomes would get very high breadths by mapped reads coming from the same strain, resulting in no reduction of the number of ‘heterozygous’ sites until a later genome was added to the dataset. For this reason, we also did an extra mapping step against only the first and last added genomes in the iterative mapping. If the number of ‘heterozygous’ sites was already lower than 10 sites per kilobase with these two genomes, the sample was considered as coinfected only by these two strains. Lastly, samples from which a *Wolbachia* genome had been assembled were also assigned the corresponding strain, independently of the competitive Illumina mapping approach.

### 2.5 Statistical Analysis of *Wolbachia* Presence and Diversity in Sister Pairs of European Butterflies

To investigate *Wolbachia* co‐cladogenesis, we built a correlation between mean host nuclear divergences and mean *Wolbachia* divergences. For *Wolbachia*, we used average nucleotide identity (ANI) as implemented in FastANI (Jain et al. 2018) as a measure of strain similarity. We obtained the mean ANI between two species in a pair by computing the mean of all pairwise comparisons of all *Wolbachia* occurrences in one species against all occurrences in the other species. Pairwise comparisons were computed both ways since ANI is asymmetric. For the hosts, we used mean gene divergences among the sister species (*d*_xy_) obtained from Ebdon et al. (2021), who investigated the relationship between divergence time and the occurrence of contact zones in European butterfly sister species pairs.

In addition, we also calculated if mean ANI between sister pairs is significantly higher than expected by chance by drawing an empirical *p*‐value from a null distribution of mean ANI values generated as follows: for each replicate, we randomly assigned the species in pairs without repeating any, obtained the mean ANI of each pair in the same way as for the true pairs, and computed their mean. With all these replicates (30,000; including the observed value), we generated a distribution of mean ANI values in random pairs, and computed how many values were greater than or equal to the observed value. This was done two times, once including strains shared between both species (which yield an ANI of 100) and once excluding them. To assess potential evidence of a role of *Wolbachia* in host speciation, we calculated if the number of strains shared by sister taxa is higher, equal, or lower than expected by chance by generating a null distribution of mean numbers of shared strains in randomised species pairs as explained above, but using a matrix of strain‐sharing instead of ANI, and counting the number of strains occurring in both species in each randomised pair. This was done three times: once with all species pairs (*N* = 36 species), once including only species pairs with at least one species infected (*N* = 26), and once including only those pairs with both species infected (*N* = 16). 30,000 replicates were computed in all three cases; since the results were consistent, we only present those considering only pairs where both species are infected.

To assess how the relationship between species pairs affects their similarity in *Wolbachia* status, we built a Bayesian logistic regression with the MCMCglmm R package (Hadfield 2010), using strain sharing (i.e., whether a sister‐species pair shares strains) as the response variable. Only the twelve pairs in which at least one of the species is infected were considered (*N* = 12). As predictor variables, we included the time since the split of the sister species, the degree of sympatry (i.e., the extent to which the distribution ranges of a sister species pair overlap), and their interaction. We fitted two models, one with split time in generations and another with split time in million years (Mya). Split time and degree of sympatry estimates were obtained from Ebdon et al. (2021). Briefly, mean nuclear genetic divergence among a sister pair was re‐scaled to split time using the *Heliconius melpomene* mutation rate (Keightley et al. 2015), and butterfly records from the Distribution Atlas of European Butterflies and Skippers (Kudrna et al. 2011) and from the collection of the Institut de Biologia Evolutiva (IBE, Barcelona, Spain) were used to compute convex hulls for each species with the recluster R package (Dapporto et al. 2013), and the degree of sympatry between sister species was computed as the fraction of the smallest of the two ranges that was also part of the other species' range. The significance of each model term was assessed based on the pMCMC, a metric provided by the MCMCglmm package, which is similar, but not identical, to the frequentist *p*‐value. Specifically, the pMCMC is defined as two times the probability that the estimated parameter is either above or below zero (whichever is smallest). For example, if the posterior distribution goes mostly over negative values, with a ‘tail’ into the positive range, then the pMCMC is two times the area of the ‘tail’ of the distribution that falls over positive values.

For all statistical analyses, the infection status of a species was derived based on our data, without considering infection reports from the literature. We took this decision because many studies are based on MLST and/or cover different geographic areas, and are thus not directly comparable to our analyses of infection status and strain identity. Analyses were performed under R version 4.3.0.

### 2.6 Comparison of *Wolbachia* and Butterfly Split Times

To compare the divergences between the *Wolbachia* strains found in a given pair and that of the butterfly species, we took the concatenated genomes representative of the strains assigned to each WGS resequencing sample and the corresponding BAM files, conducted variant‐calling with FreeBayes (Garrison and Marth 2012), and generated consensus sequences for each of the strains. Each consensus sequence therefore represents a *Wolbachia* ‘isolate’ from a given butterfly specimen. ANI estimates were generated for all possible pairs of consensus sequences and transformed into genetic distances (1 − ANI/100). Then, genetic distance was corrected with the Jukes–Cantor formula (Jukes and Cantor 1969) and translated to divergence times using the maximum and minimum possible substitution rates estimated by Richardson et al. (2012). Specifically, we took the upper bound of the credible interval for the rate of noncoding DNA positions (1.5 × 10^−9^ substitutions/site/host generation) and the lower bound of the credible interval for the rate of first and second codon positions (2.76 × 10^−10^).

We also calculated mitochondrial split times by extracting the sequences of the protein‐coding genes (PCGs) from the assembled mitogenomes, computing their divergences, correcting them with the Jukes–Cantor formula, and translating them to divergence times using the widely accepted rates of Brower (1994) (2.3% uncorrected pairwise distance per million years) and Quek et al. (2004) (1.5% uncorrected pairwise distance per million years).

For all possible comparisons of *Wolbachia* strains in butterfly species within the same genus, we obtained the maximum and minimum divergence time estimates considering all comparisons of the strains in question, and compared those ranges to the butterfly split times from Ebdon et al. (2021). This was done considering comparisons of the samples assigned to the same strain in the same butterfly species (intraspecific intrastrain), of different strains in the same butterfly species (intraspecific interstrain), of the same strain in different species (interspecific intrastrain), and of different strains in different species (interspecific interstrain). For each comparison, the split times were considered congruent if: (1) the smallest of the two ranges being compared completely overlapped with the largest of the two or (2) the midpoint of the largest range was within the smallest range (i.e., if the midpoint was older than the youngest point of the small range, but younger than the oldest point of the small range). Otherwise, the *Wolbachia* split was considered older if its midpoint was larger and younger if it was smaller.

### 2.7 Screening for Cytoplasmic Incompatibility and Male‐Killing Candidate Loci

To determine whether the detected strains could cause, or have previously caused, CI and MK, we screened for known loci involved in these phenotypes (Beckmann et al. 2017; Le Page et al. 2017; Perlmutter et al. 2019). To do so, we took the reference sequences of candidate CI loci cinA/cinB and cidA/cidB, and the candidate MK loci *wmk* from the genomes of the strains used for the experimental validation of such loci (Table S6) and used them as queries in a TBLASTN (Altschul et al. 1990) search against the *Wolbachia* genomes newly assembled in this study. Hits with *e*‐values < 10^−20^ and query coverage > 60% were kept for a BLASTX search against the proteomes of the strains in which the CI/MK loci were characterised (e.g., hits of the *wmk* locus against the *B. hecate* strain were BLASTX searched against the proteome of wMel strain from *Drosophila melanogaster* ). Sequences whose first BLASTX hit against the proteomes corresponded to the same loci that originated the initial TBLASTN hit were then compared with the structural annotation of the de novo *Wolbachia* genomes to check their concordance with the predicted genes, and hits that did not match the predicted proteins were discarded. In the case of CI loci, which consist of a putative toxin–antitoxin system where both genes are adjacent in the genome, consecutive placement of the complementary loci was also inspected.

The software versions and parameters used for each program are available in Table S7. When appropriate, analyses were run using GNU Parallel (Tange 2022).

## 3 Results

### 3.1 Overview of the Assembled Genomes

Out of 26 genomic reference specimens, we assembled 12 high‐quality *Wolbachia* genomes (Table 1). Genome sizes ranged from 1,315,265 bp (strain from *Pontia edusa*) to 1,926,985 bp (strain from *Thymelicus acteon*), with an average of 1,499,202 bp. The GC content ranged from 0.339 to 0.341, with an average of 0.340. All genomes had a BUSCO completeness above 90% and duplication below 5%. The number of coding sequences (CDS) predicted by Prokka (Seemann 2014) ranged from 1204 (*P. edusa*) to 1917 (*T. acteon*), with an average of 1430 (Table 1).

**TABLE 1:** Quality metrics of the *Wolbachia* genomes assembled in this study.

Host species	*N* contigs (> 1 kbp)	Span (> 1 kbp)	*N*50	Longest scaffold	GC%	BUSCO C%	BUSCO M %	No. of CDS	Mean protein length	No. of rRNA	No. of tRNA
*Brenthis hecate*	1	1,416,212	1,416,212	1,416,212	0.340	99.8	0.2	1350	308.82	3	37
*Colias alfacariensis*	1	1,464,674	1,464,674	1,464,674	0.341	99.5	0.2	1420	303.80	3	37
*Colias hyale*	1	1,652,361	1,652,361	1,652,361	0.340	99.4	0.3	1546	311.90	3	35
*Erebia ligea* EE_932	2	1,615,307	1,320,503	1,320,503	0.341	99.4	0.3	1566	300.95	3	35
*Erebia ligea* EL_949	9	1,472,687	738,288	738,288	0.340	98.3	0.3	1437	292.17	3	36
*Gonepteryx cleopatra*	1	1,419,660	1,419,660	1,419,660	0.340	99.4	0.6	1331	312.94	3	35
*Iphiclides podalirius*	1	1,315,434	1,315,434	1,315,434	0.340	99.5	0.2	1300	294.04	3	35
*Iphiclides feisthamelii*	2	1,357,406	1,271,026	1,271,026	0.340	98.4	0.5	1273	309.42	3	36
*Lasiommata petropolitana*	1	1,466,526	1,466,526	1,466,526	0.341	99.7	0.3	1383	314.63	3	36
*Polyommatus eros*	1	1,567,905	1,567,905	1,567,905	0.341	99.4	0.3	1437	320.78	3	36
*Pontia edusa*	2	1,315,265	1,268,042	1,268,042	0.339	99.7	0.3	1204	320.01	3	35
*Thymelicus acteon*	7	1,926,985	552,656	664,073	0.341	96.9	2.8	1917	292.85	3	34

*Note:* There are two assemblies from *Erebia ligea*, as we had long read data for two specimens of this species.

### 3.2 Phylogenetic Inference and Co‐Phylogenetic Analysis

**FIGURE 1:**
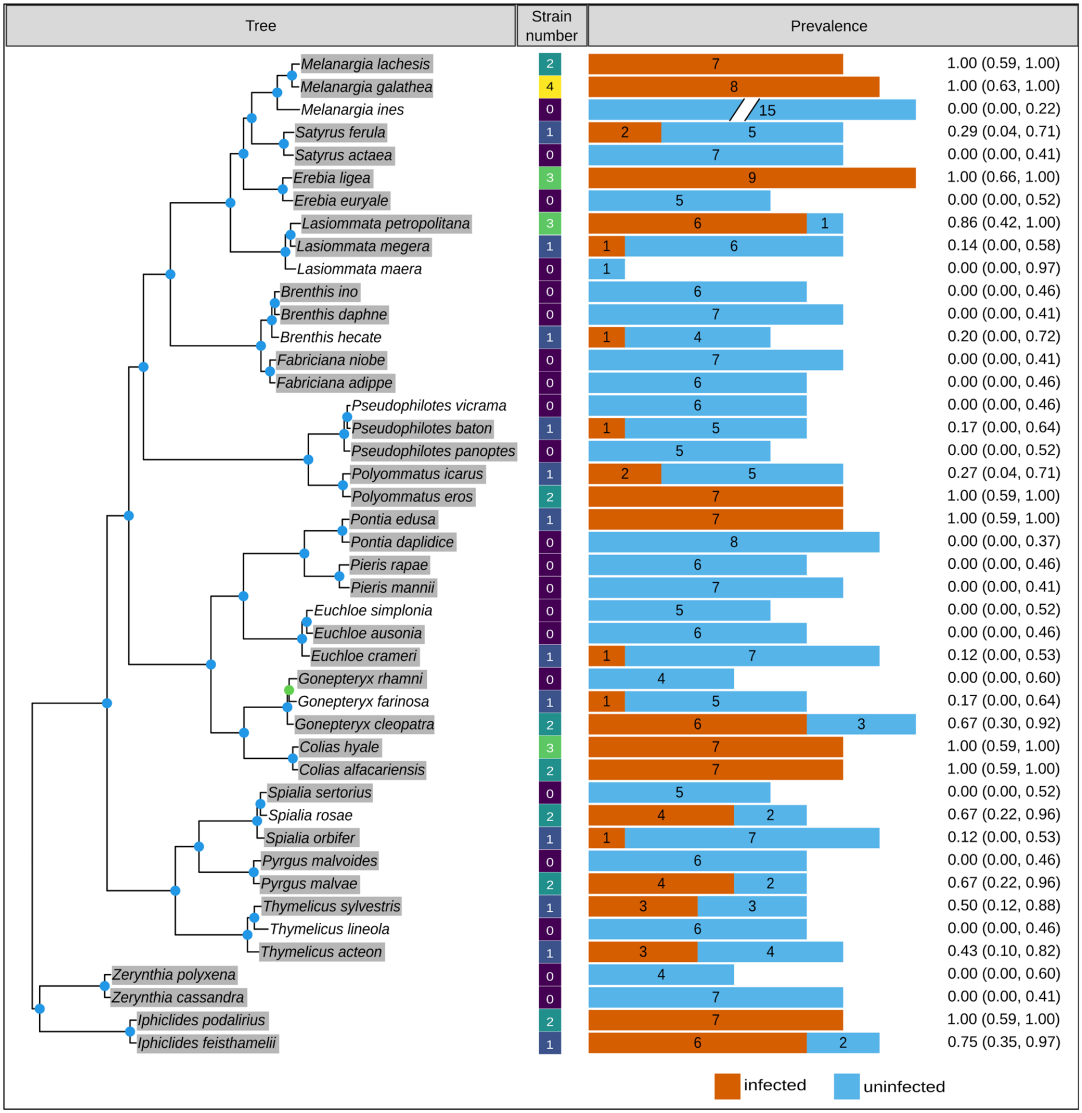
Butterfly phylogeny alongside *Wolbachia* prevalence in each of the butterfly species. Species highlighted in grey correspond to the sister species pairs considered for the analyses of strain sharing and strain similarity. Internal nodes in the phylogeny are coloured according to their bootstrap support; blue nodes had a support of 100% for both UFBoot and SH‐aLRT. The middle column indicates the number of *Wolbachia* strains identified in each species. The length of the orange (left) and blue (right) bars are proportional to the number of infected and uninfected samples, respectively, which are indicated with a number over each bar. To improve visibility, the bar of *M. ines* is not to scale. The numbers at the right of the barplot indicate the mean and 95% confidence intervals of the *Wolbachia* prevalence in that species.

The butterfly nuclear phylogeny was highly supported, with both ultra‐fast bootstrap (UFBoot) and SH‐aLRT values of 100% for all branches except for the branch joining *Gonepteryx rhamni* and *G. farinosa* (Figure 1). The relationships among families, subfamilies, and genera agreed with those already established (Espeland et al. 2018; Wiemers et al. 2020). The majority of relationships among species pairs and trios also agreed, with the exception of two trios: our phylogeny groups *Gonepteryx rhamni* as sister to *G. farinosa* instead of *G. cleopatra* and *Spialia rosae* as sister to *S. sertorius* instead of *S. orbifer*. Nonetheless, even if a small minority of the pairs defined in Ebdon et al. (2021) are not ‘true’ sister species, they are closely related, with hybrids observed in the wild for some of them (Ebdon et al. 2021). Since we do not expect the definition of pairs to influence our analyses, we analysed *Wolbachia* strain sharing for the same butterfly host pairs studied by Ebdon et al. (2021), which allowed us to use the same measures of host range overlap.

Most nodes in the butterfly mitochondrial phylogeny were also highly supported by both UFBoot and SH‐aLRT values, except the node grouping *Melanargia* and *Satyrus* (9.5/49), the clade conforming to the Pierinae (86.3/84), and a clade formed by Pieridae and Lycanidae (42/56), which differs from currently accepted relationships among families (Espeland et al. 2018; Wiemers et al. 2020). This appears to be a long‐branch attraction (LBA) artefact, as the branches leading to Lycaenidae and Pieridae are long, and our sampling is sparse at a subfamily level and also lacks Riodinidae, the sister family to Lycaenidae.

**FIGURE 2:**
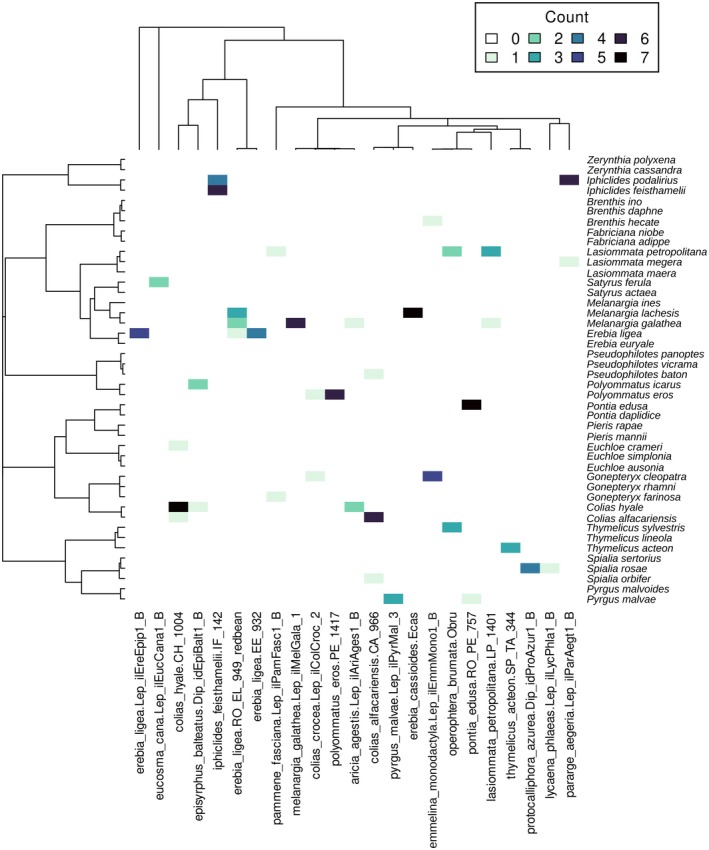
Interaction matrix between *Wolbachia* strains (columns) and butterfly hosts (rows). Each cell in the matrix is coloured according to the number of specimens of the butterfly host that harboured the corresponding strain. The strains are ordered according to their phylogenetic relationships, shown at the top of the figure. The butterfly hosts are ordered based on the nuclear phylogenetic relationships, shown at the left of the figure.

The complete *Wolbachia* phylogeny containing strains from all supergroups (*N* = 195) was well supported (Figure S2) and agreed with previous results (Vancaester and Blaxter 2023). As all strains in our samples belonged to supergroup B, we built a second phylogeny of that supergroup (*N* = 88) with a higher number of SCOs. This phylogeny was also highly supported, with most branches having UFBoot and SH‐aLRT support values of 100% (Figure 2). The relationships among strains were generally consistent with the complete phylogeny, with some exceptions, such as the placement of the *Colias hyale* strain or the relationships within the clade containing the *Pammene fasciana* strain at the base (see Figure 2; Figure S2).

There was no clear topological congruence between the *Wolbachia* and the butterfly nuclear (Figure 2; Figure S3) or mitochondrial (Figures S4 and S5) phylogenies. Some sister species pairs (*Gonepteryx* spp. and *Colias* spp.) shared strains, but these strains were also found in other genera, which is suggestive of horizontal transfer (since the multiple secondary losses required to explain this pattern are unlikely; Figure 2). Therefore, the butterfly and *Wolbachia* phylogenies cannot be reconciled without introgressive or horizontal symbiont transfer events. The Random TaPas analysis with the nuclear phylogeny gave a normalised Gini coefficient of 0.682 ± 0.017, which is considered a low co‐phylogenetic signal (Balbuena et al. 2020). The analysis with the mitochondrial phylogeny yielded a similar coefficient (0.694 ± 0.009).

### 3.3 *Wolbachia* Presence in Each Species Pair

Of the 44 butterfly species analysed, 23 had at least one specimen infected with *Wolbachia*, giving a sample incidence of 52% (Figure 1; Table S8). Between these infected species, sample prevalence ranged from 14% to 100%, with a mean and standard deviation of 61.01% ± 35.75% considering only infected species. These sample prevalences are not accurate estimates of the species‐level prevalence, but are at most indicative as to whether *Wolbachia* is likely rare/absent or very frequent in a species. Most infected butterfly species had one or, less often, two strains, with the exception of *Colias hyale* and *Lasiommata petropolitana*, which were infected by three strains, and *Melanargia galathea*, which had four (Table S8). Although we found no obvious geographic pattern of infection, changes in strain distribution in some host species seem to match geographic barriers such as mountain ranges, ocean straits, or islands (Table S1). For example, the only infected individual of *Brenthis hecate* is the sample from the Iberian Peninsula, which is isolated from the rest of the distribution area by the Pyrenees; and the only uninfected specimens of *Iphiclides feisthamelii* are from Morocco.

Twenty of the 44 species are, to our knowledge, screened for *Wolbachia* for the first time. Of the remaining species, seven have been previously reported as infected but were uninfected in our dataset, while one has been reported as uninfected but was infected in our dataset; the remaining 16 species agreed with previous reports (see Table S8 for details). In six of the 18 species pairs, both species were infected; in eight pairs, only one species was infected, and four pairs showed no signs of infection in either species (Table S9). Of the pairs where both were infected, three shared some strains between both species, while all six had at least one strain that was unique to one species in the pair.

### 3.4 Statistical Analysis of *Wolbachia* Presence and Diversity in Sister Pairs of European Butterflies

#### 3.4.1 Sharing of Strains Across Species

**FIGURE 3:**
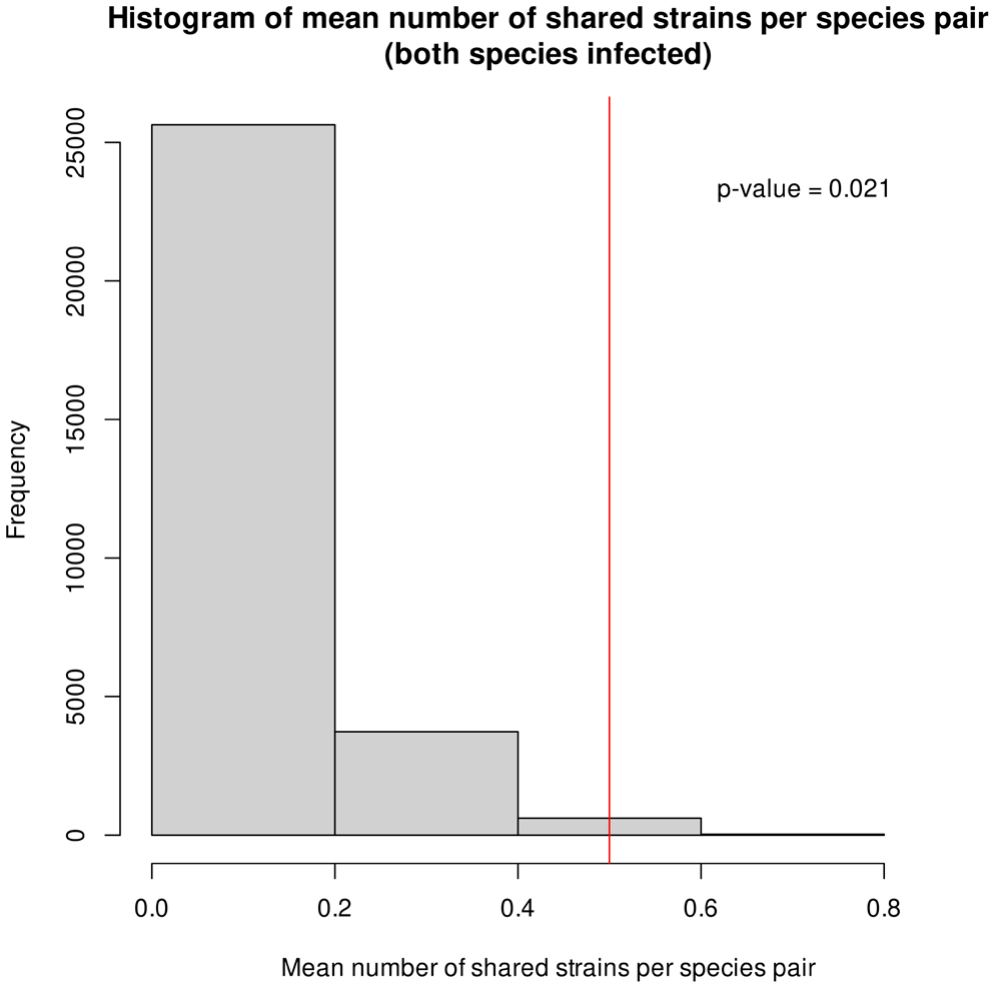
Results of the randomization of species pairs. The histogram indicates the distribution of the mean number of shared strains for the randomised species pairs, so that the frequency (*y*‐axis) indicates how many of the random replicates had a given mean number of shared strains; the red line indicates the observed value of the true sister‐species pairs.

We found no significant correlation between butterfly nuclear divergence (*d*_xy_) and *Wolbachia* genome similarity (ANI; Figure S6). The mean number of strains shared within host species pairs was significantly higher than that expected for random species pairs (*p* < 0.05; Figure 3). The mean ANI for true host pairs was close to the expectation when the randomization procedure did not consider shared strains (Figure S7A), while it was shifted towards higher values (but not significant) when considering shared strains (whose ANI is 100; Figure S7B), in concordance with the finding of higher strain sharing in true pairs.

In the Bayesian models of strain sharing, when host split times were measured in number of generations, we found that older host split times significantly reduced the probability of strain sharing (*p* < 0.05), and this effect was larger for more sympatric host pairs (i.e., a significant interaction term, *p* < 0.05; Figure S8; Table S10). However, when split times were measured in million years, the effect sizes were smaller, and only the interaction term was significant (Figure S9; Table S10).

#### 3.4.2 Comparison of *Wolbachia* and Butterfly Split Times

**FIGURE 4:**
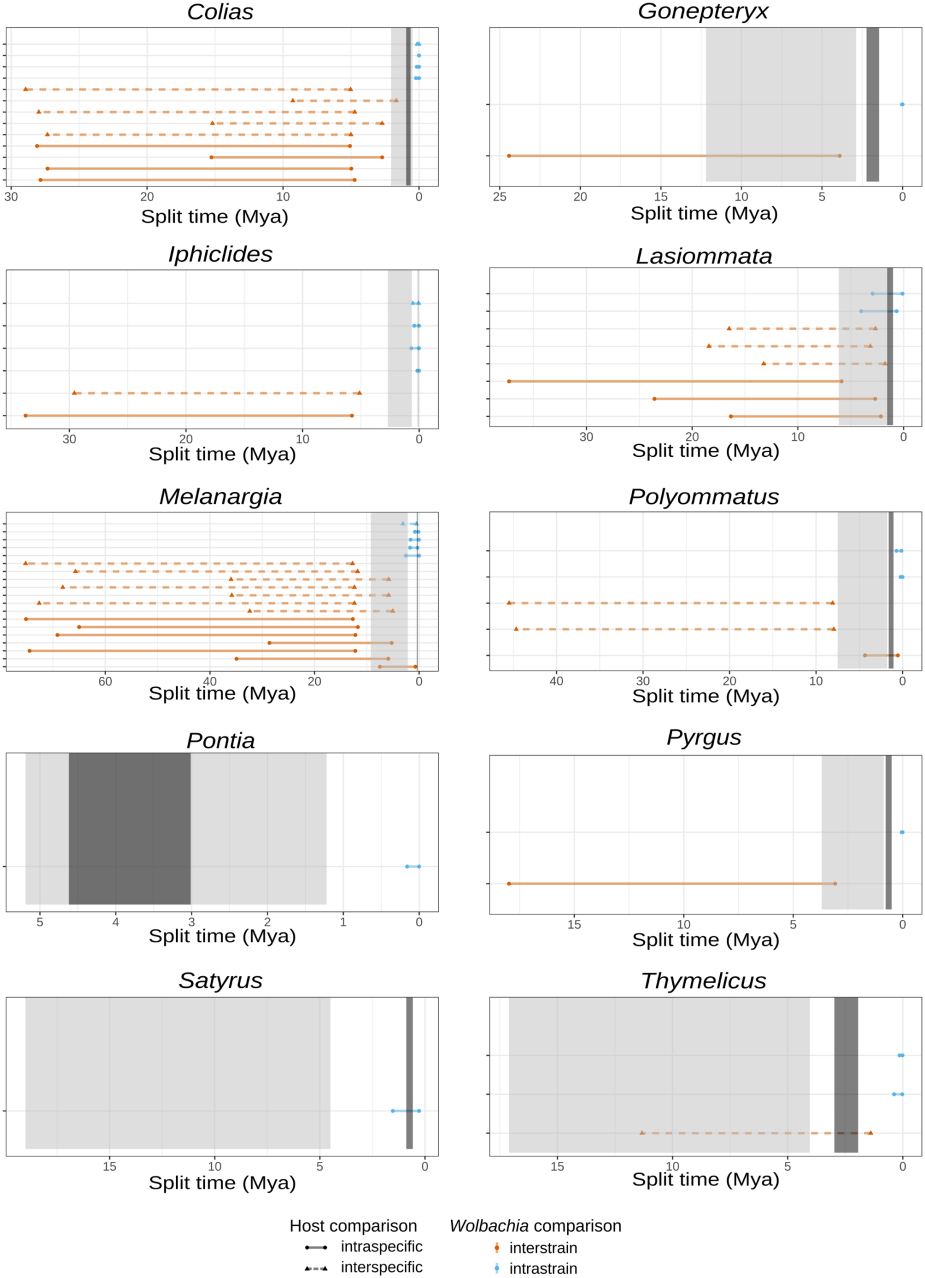
Ranges of split times between *Wolbachia* isolates found in different butterfly specimens. The different strain comparisons are presented along the *y*‐axis, grouped according to whether the compared *Wolbachia* isolates correspond to the same strain (intrastrain; orange) or different strains (interstrain, blue) and whether they are found in the same butterfly species (intraspecific; dashed lines with triangle tips) or to different, putatively sister, butterfly species (intraspecific; solid lines with circular tips). The light grey rectangles correspond to the confidence intervals for the split times of the sister butterfly species based on nuclear data estimated in Ebdon et al. (2021); the dark grey rectangles indicate the range of mitochondrial split times estimated from the mitogenomes assembled in this study.

For most species pairs, mitochondrial split time estimates were younger than the corresponding nuclear estimates (Figure 4). At an intraspecific level, most *Wolbachia* splits were younger than the corresponding host nuclear sister split times, a result that is consistent with vertical transmission (Figure 4; Table S11). There was one exception in *Melanargia*, in which the strain divergence was older than the sister hosts' mitochondrial split time, suggesting horizontal transfer. In contrast, most interstrain comparisons had older split times than the corresponding hosts, suggesting horizontal acquisition of at least one of the strains. There was one exception in *Polyommatus*, which had a recent split time congruent with those of the host sister pair and therefore with vertical transmission of the two strains.

At an interspecific level, two intrastrain comparisons showed younger split times than those of the butterfly hosts (both mitochondrial and nuclear), indicating horizontal transfer, whereas another (in *Iphiclides*) was more recent than the nuclear split time, but congruent with the mitochondrial split time, suggesting introgressive transfer of the strain (Figure 4; Table S11). All interstrain comparisons had older *Wolbachia* split times than host split times, indicating distant, horizontally transferred strains.

### 3.5 Presence of CI and MK Loci in *Wolbachia* Genomes

All newly assembled *Wolbachia* genomes contained candidate sequences for the cytoplasmic incompatibility loci (Table 2; Table S12). All strains showed at least one set of consecutive cifA and cifB candidates, with most having one or two sets but going up to six in the strain from *Colias hyale*. All genomes except the strain from *Pontia edusa* included matches to the *wmk* male‐killing factor, with a mean of two candidates but ranging up to six in the strain from *Thymelicus acteon* (Table 2; Table S12). In all cases, the candidate sequences overlapped with annotated genes predicted by Prokka, and in most cases, had the same open reading frame and no stop codons (Table S12).

**TABLE 2:** Count of putative homologues for the loci involved in cytoplasmic incompatibility (cifA + cifB) or in male killing (wmk) in the de novo *Wolbachia* genomes assembled in this study.

Genome	Consecutive cifA + cifB	Non‐consecutive cifA	Non‐consecutive cifB	wmk
w.brenthis_hecate.BH_1412	1	1	1	3
w.colias_alfacariensis.CA_966	1	0	0	2
w.colias_hyale.CH_1004	6	0	1	1
w.erebia_ligea.EE_932	2	0	0	4
w.erebia_ligea.RO_EL_949	1	1	0	3
w.gonepteryx_cleopatra.GR_164	1	3	1	3
w.iphiclides_feisthamelii.IF_142	2	0	0	2
w.iphiclides_podalirius.IP_504	1	0	0	1
w.lasiommata_petropolitana.LP_1401	3	0	0	1
w.polyommatus_eros.PE_1417	2	2	2	2
w.pontia_edusa.RO_PE_757	2	0	0	0
w.thymelicus_acteon.SP_TA_344	3	1	2	6

*Note:* The column ‘consecutive cifA + cifB’ refers to the count of consecutive cifA and cifB that were recovered (i.e., the count of putative cifAB operons); while the columns ‘non‐consecutive cifA’ and ‘non‐consecutive cifB’ refer to the count of putative cifA and cifB homologues, respectively, that did not appear to be adjacent to a cifB/cifA homologue. The column ‘wmk’ refers to the count of putative wmk homologues.

## 4 Discussion

This study investigated *Wolbachia* transmission among recently diverged sister species pairs of European butterflies, as well as the presence of CI and MK loci in the genomes of the associated *Wolbachia* strains. Sister‐species pairs shared *Wolbachia* strains more often than random pairs, and the probability of sharing strains was positively correlated with range overlap, that is, the degree of sympatry. In addition, the split times between *Wolbachia* strains often predated host divergence, although in some cases, mitochondrial and *Wolbachia* divergences were almost congruent. Our results suggest that horizontal (and, to a lesser extent, introgressive) transfer is frequent even between recently speciated taxa. Additionally, most of the newly assembled *Wolbachia* genomes contain putative homologues of the CI and MK loci, suggesting they either are or were previously able to induce such effects.

### 4.1 Introgressive and Horizontal *Wolbachia* Transfer Among Sister Pairs

Contrary to what would be expected under strict co‐cladogenesis, we found no significant correlation between *Wolbachia* ANI and host nuclear divergence (*d*_xy_). This suggests either that co‐cladogenesis does not occur frequently or that the *Wolbachia* strains involved are quickly lost or replaced, erasing co‐phylogenetic patterns even in very young host species.

Sister species hosted the same strains more often than non‐sister species, which is compatible with both secondary acquisition via introgressive transfer and co‐cladogenesis. In contrast, *Wolbachia* strains from sister pairs did not have a significantly higher ANI compared to random species pairs, indicating that, when sister species have different strains, these are not, on average, more closely related than different strains occurring in non‐sister species pairs. This may be explained by a preponderance of *Wolbachia* horizontal transfer from relatively distant hosts, which would make strains found in sister pairs no more closely related than strains found in random species pairs. It must be noted that both the ANI and the strain‐sharing results are susceptible to our sampling scheme, in which we prioritised sampling host species at the cost of sampling effort within species (6–7 specimens in most cases). With denser sampling, strain sharing is bound to increase, but whether this would increase more among host sister species pairs (which would reinforce our conclusions) than random pairs remains to be determined.

Regardless of the sampling uncertainty, the Bayesian model of strain sharing revealed a significant interaction effect between host split time and degree of sympatry on the probability of sharing *Wolbachia* strains. This also suggests that introgressive and/or horizontal transfer between host sister taxa is frequent, as range overlap facilitates the transfer of *Wolbachia* by providing more opportunities for species to hybridise or to exchange symbionts via shared resources or natural enemies. Previous studies have also stressed the importance of physical contact and, consequently, of sympatry, for horizontal transfer (Gerth et al. 2013; Schuler et al. 2013).

Lastly, most estimates of *Wolbachia* interstrain divergence were substantially older than both nuclear and mitochondrial estimates of host divergence, suggesting that horizontal transfer is prevalent. On the other hand, comparisons within a strain and within the same host species were compatible with vertical transmission. Comparisons of shared strains across species in a pair were generally younger than the mitochondrial divergence of their hosts, which is compatible with independent acquisition via horizontal transfer. There was one exception in *Iphiclides*, in which mitochondrial and symbiont split times were congruent and thus compatible with recent introgressive transfer, as expected based on Gaunet et al. (2019).

It is worth considering several factors that affect our results. First of all, mitochondrial split times were lower than the corresponding nuclear estimates in most cases. This can be due to the lower effective population size for mitochondrial DNA, which (assuming neutral evolution and balanced sex ratios) is ¼ that of the nuclear DNA, resulting in more recent coalescence times (Allio et al. 2017). On the other hand, past mitochondrial introgression events (not necessarily related to current *Wolbachia* infections) could also cause the more recent mitochondrial split times.

In addition, the different molecular clocks we have used to date mitochondrial, nuclear, and *Wolbachia* divergence are associated with large (and largely unknown) errors. Since we used a wide range of possible substitution rates for *Wolbachia*, and since in most cases symbiont and host split times are incongruent, obtaining estimates based on more accurate molecular clocks would most likely widen the gap between the estimated split times even more. In addition, the *Wolbachia* evolutionary rates of Richardson et al. (2012) likely overestimate the actual rates, as they are based on intraspecific lineages in *Drosophila melanogaster* and therefore include mildly deleterious mutations that are unlikely to fix and contribute to the substitution rate. Calibration with lower mutation rates would push interstrain divergence times back, making them even less congruent with host divergence. In the case of intrastrain comparisons, some older *Wolbachia* split times may become congruent with the mitochondrial host divergence estimates, which would support introgressive transfer and help explain the observed discrepancy between mitochondrial and nuclear split times (although other factors can explain this, as discussed above). Indeed, there is evidence of introgressive transfer of *Wolbachia* in the butterfly genera *Plebejus* (=*Lycaeides*; Shastry et al. 2022) and *Erebia* (Lucek et al. 2021). Given that some of the studied sister pairs are known to hybridise (Ebdon et al. 2021), introgressive transfer is plausible and may be more prevalent than we have inferred. However, despite the uncertainty of molecular clock calibrations, our results support horizontal transfer as a major mode of *Wolbachia* acquisition.

Our results add to the growing body of evidence for introgressive and horizontal transfer of *Wolbachia*, generally at larger phylogenetic scales (both in butterflies, Duplouy et al. 2020; Zhao et al. 2021; and arthropods in general, Turelli et al. 2018; Vancaester and Blaxter 2023; but see Scholz et al. 2020). We complement these studies by showing that horizontal transfer is also frequent over intermediate timescales, involving closely related host species. In agreement with our results, a recent study of neotropical *Spicauda* butterflies found distantly related strains in closely related host species, suggesting horizontal or introgressive transfer (Ribeiro et al. 2025). Therefore, high *Wolbachia* turnover mediated by horizontal transfer appears to be a global feature in Lepidoptera, occurring in both temperate and tropical regions. In agreement with this, recent theoretical work suggests that clade selection favours the long‐term persistence of CI‐inducing strains through horizontal/introgressive transfer over non‐CI inducing strains, which are less likely to successfully invade new hosts (Turelli et al. 2022).

Determining the exact turnover rate is challenging, as we are limited to observing current infections. Nonetheless, we can derive an estimate based on a simple model: assuming an equal rate of symbiont acquisition and loss and counting the number of strain differences across all 18 pairs of butterfly sister species and the total host divergence time (in generations) yields a turnover rate of 0.16 acquisition or loss events per million host generations, which (given 1–3 butterfly generations per year) translates to one event every 6.1–2.1 million years (My; see Data S1). In comparison, Bailly‐Bechet et al. (2017) estimated an average duration of 7 and 9 My for infected and uninfected phases, respectively, that is, a lower rate of turnover. One possibility is that butterflies genuinely have a higher *Wolbachia* turnover than other arthropods (note that our calculation ignores the possibility of repeated loss and gain events and so is a lower bound). Alternatively, the dynamics in small and isolated archipelagos (as sampled by Bailly‐Bechet et al. 2017) may be slower than those of continental areas, as there may be fewer opportunities for new *Wolbachia* strains to invade. Additionally, denser sampling may affect the observed proportion of shared strains and therefore our estimate of turnover rate. Further studies are required to determine the causes of these different rates.

Although less common, there is also evidence of co‐cladogenesis in some arthropods (Gerth et al. 2013; Raychoudhury et al. 2009). In bedbugs and filarial nematodes in particular, where *Wolbachia* is an obligate mutualist residing in specialised bacteriocytes, there is a high level of congruence among host and symbiont phylogenies (Lefoulon et al. 2016; Balvín et al. 2018). Furthermore, Gerth and Bleidorn (2016) suggest that, in the case of *Nomada* bees, congruence is favoured by their high degree of specialisation, which limits opportunities for symbiont transfer. Thus, although only a fraction of *Wolbachia* strains infecting butterflies have been characterised in detail, the lack of co‐cladogenesis is congruent with a generally parasitic type of interaction in the Papilionoidea superfamily as a whole.

### 4.2 Widespread Occurrence of CI and MK Loci

In agreement with the view of *Wolbachia* as a reproductive parasite, we found putative homologues of the CI loci in all the newly assembled genomes, as well as putative homologues of the MK locus *wmk* in most of them. Consistent with previous screens of cif genes in *Wolbachia* (Martinez et al. 2021; Tan et al. 2024), most strains showed one or two sets of consecutive cifA/B genes. It therefore seems likely that at least part of the recovered strains can, or previously could, induce CI and/or MK in an appropriate host genetic background and environment. It must be kept in mind that these are putative homologues, whose detection does not imply that CI or MK are currently expressed (indeed, strong MK would likely have been reported). Martinez et al. (2021) found a significant association between the presence of cifA/cifB pairs without disrupting mutations and the reported occurrence of CI. The cif candidates reported here overlap with annotated proteins and do not contain stop codons, which suggests that they are functional, but it is still possible that some of them are not; for example, they may not be expressed and/or they may have other mutations in key amino acid residues. Indeed, degradation of the CI loci has been previously reported (Lindsey et al. 2018; Meany et al. 2019). In addition, hosts may have evolved resistance (Hornett et al. 2006). The number of cif copies has also been suggested to positively correlate with the strength of CI (Le Page et al. 2017), so strains with a single cif set (5 out of 12 in our case), if functional, may present a weak CI phenotype. Regardless, the ubiquity of CI and MK loci is consistent with the lack of phylogenetic congruence and the evidence for horizontal transfer, and also supports the current view of *Wolbachia*‐mediated sweeps as an explanation for the mitonuclear discordance observed in some butterfly taxa (Gaunet et al. 2019; Kodandaramaiah et al. 2013; Shastry et al. 2022), as CI would allow the symbiont to quickly spread through a population, dragging the mitochondrial genome with it.

Turelli et al. (2022) hypothesised that CI favours the spread and persistence of *Wolbachia* strains regardless of whether they have mutualistic effects, because CI (1) allows *Wolbachia* to achieve higher prevalence, facilitating the spread to new hosts by providing more opportunities for horizontal transfer and (2) increases the expected persistence time in a host population. However, the same authors also suggest that natural selection should favour mutualistic effects in *Wolbachia*, and that deleterious strains that reduce host fecundity can only spread into new hosts with low effective population sizes. To our knowledge, there is only one potential case of *Wolbachia* mutualistic effects in Lepidoptera described to date: in moths of the family Gracillariidae the endosymbiont is thought to be involved in the production of green patches in senescent leaves necessary for successful development of the larvae (Kaiser et al. 2010). Given that other *Wolbachia* mutualists display higher phylogenetic congruence with their hosts, future studies may investigate co‐phylogenetic patterns and presence of CI and MK loci in strains infecting these moths.

Given the widespread occurrence of CI and MK candidate loci and the high *Wolbachia* turnover rate in butterflies, determining *Wolbachia* infection status may also matter in a conservation context. The spread of *Wolbachia* can reduce both census and effective host population sizes (Nice et al. 2009) as well as the average fitness (Charlat et al. 2002). In addition, small host populations are also more susceptible to *Wolbachia* invasion in the first place (Turelli et al. 2022). Since both reproductive manipulation phenotypes and host shifts appear to be frequent, *Wolbachia* poses a potential risk to endangered butterfly populations.

Despite the potential negative effects of *Wolbachia* for endangered populations, the infection status and (for infected species) *Wolbachia* phenotype remain unknown for the majority of the European butterfly fauna. Future efforts should be directed at filling this knowledge gap, particularly for species of conservation concern and phylogenetically or ecologically related taxa. It will be important to screen *Wolbachia* strains for the presence of CI and MK loci, since resistance evolution in already infected host species/populations may make such strains appear as non‐CI/MK‐inducing while still being able to have CI and MK effects once transferred to new hosts (Hornett et al. 2006).

## 5 Conclusions

We have conducted a taxonomically comprehensive screening of *Wolbachia* in the European butterfly fauna, with a focus on recently diverged sister pairs. Our results agree with previous estimates of prevalence and incidence, and match current evidence of horizontal and introgressive *Wolbachia* transfer at large evolutionary timescales. However, we also find that both phenomena are frequent at the shorter timescales over which speciation processes unfold. This suggests that *Wolbachia* turnover is higher than has been inferred from studies of more distantly related hosts. In addition, CI and MK loci may be widespread in *Wolbachia* strains associated with butterflies, supporting the view that this bacterial endosymbiont is a reproductive parasite and a driver of mitonuclear discordance, although mutualistic effects cannot be ruled out and should be explored further. Given the apparent ease of *Wolbachia* acquisition in butterflies, efforts should be made to determine the infection status of butterfly populations, particularly threatened ones, and understand the potential negative effects on host population dynamics.

## Author Contributions

Conceptualization: R.V., K.L., G.T. and D.R.L. Data curation: K.L. and E.T.‐D. Formal analysis: E.T.‐D. Funding acquisition: R.V., K.L. and A.H. Investigation: E.T.‐D. Methodology: E.T.‐D., K.L. and D.R.L. Project administration: R.V., K.L., D.R.L. and G.T. Software: E.T.‐D. and D.R.L. Resources: K.L., D.R.L., R.V., G.T. and A.H. Supervision: R.V., K.L., D.R.L. and G.T. Validation: E.T.‐D. and D.R.L. Visualisation: E.T.‐D. and D.R.L. Writing – original draft: E.T.‐D. Writing – review and editing: R.V., K.L., D.R.L., G.T., A.H. and E.T.‐D.

## Ethics Statement

Field sampling of butterflies was conducted in compliance with the School of Biological Sciences Ethics Committee at the University of Edinburgh and the European Research Council ethics review procedure. When required, permits to collect specimens were obtained from the corresponding authorities: Greece (165948/157; 110097/1299; 112441/1860), Spain (Catalonia [SF/276; SF/279–SF/285; SF/346–SF/357; SF/151–173; SF/0048/2019; SF/0210], Andalusia [SGYB‐AFR‐CMM; PNSN/JSG/JCMK/MJB; 327/490; 328/490; PNSN/JSG/JCML/MCF; ENSN/JSG/BRL/MCF; SGYB/AF/DBP], Castilla‐La Mancha [223064; 463056; 179851], Castilla y León [EP/CYL/123/2008; EP/CyL/291/2009; EP/AV/418/2009; EP/CYL/10/2012; EP/CyL/311/2015; EP/CyL/138/2016], Aragón [INAGA 500201/24/2015/1589], Asturias [2017/007323], Murcia [AUF/2017/0071]), Italy (00005368; 0012493; 0012131), Poland (NB‐0604.42/2016; DU‐2590/2016/ag).

## Conflicts of Interest

The authors declare no conflicts of interest.

## Supporting information


**Data S1:** mec70125‐sup‐0001‐DataS1.zip.

## Data Availability

The following *Wolbachia* read datasets are available at the European Nucleotide Archive (ENA) at EMBL‐EBI under the following BioProject accessions: PRJEB49202: *Wolbachia* read datasets for *Brenthis ino*, PRJEB56310: *Wolbachia* read datasets for *Brenthis ino* and *B. daphne*, PRJEB62818: *Wolbachia* read datasets for *Brenthis hecate* and *Fabriciana adippe*, PRJEB51340: *Wolbachia* read datasets for *Iphiclides podalirius*, PRJEB76171: *Wolbachia* read datasets for *Iphiclides podalirius* and *I. feisthamelii*, PRJEB83091: *Wolbachia* read datasets for the remaining species. Sample metadata, including the infection status of each sample, is available in Table S1. The assembled *Wolbachia* genomes are available at ENA under BioProject PRJEB83091 (accessions GCA_966215945, GCA_966217225, GCA_966217105, GCA_966213895, GCA_966216895, GCA_966216975, GCA_966216545, GCA_966216375, GCA_966213985, GCA_966214045, GCA_966214855, and GCA_968179995). The assembled and annotated mitogenomes are available at GenBank (accession numbers PX316417 to PX316453). The scripts required to replicate the analyses of this paper are available at Zenodo with the following link: https://doi.org/10.5281/zenodo.15593199.
