# Supplementary material for: Wolbachia Host Shifts and Widespread Occurrence of Reproductive Manipulation Loci in European Butterflies
Source: Mol Ecol. 2025 Oct 8;34(21):e70125. doi: 10.1111/mec.70125 (PMC12573729; doi:10.1111/mec.70125)
Supplement: Supplementary file 1 — Data S1: mec70125‐sup‐0001‐DataS1.zip. [file MEC-34-e70125-s001.zip › mec70125-sup-0001-DataS1/mec70125-sup-00011-DataS1.pdf]

Supporting Information 1

Modelling *Wolbachia* turn-over in butterfly species pairs

A simple model of a single state transition per branch

The simplest possible model of strain sharing across sister species assumes that *Wolbachia* is lost and gained at some rate μ per generation (we assume that species in each pair have the same generation time). For large divergence time T, it becomes unrealistic to ignore the probability of multiple transitions in infection state. Considering gain and losses (both occurring at rate μ) along both branches, a pair can only differ in infection status if an odd number of state transitions occurs along one of the two branches. The probability of seeing an even number of events given that the number of events in time T (i.e. along one of the two branches) follows a Poisson distribution is:

In[∘]:= $P_{even} = 1/2\left(1 + e^{-T\mu}\right);$

In[∘]:= $P_{odd} = 1/2\left(1 - e^{-T\mu}\right);$

In[∘]:= $\text{Simplify}[P_{even} + P_{odd}]$

Out[∘]= 1

If we denote the probability of the ancestor being uninfected as p_0 (and the probability of the ancestor being unaffected p_1), the probabilities of the three possible infection states in a species pair (P_0 none of the two species has *Wolb*, P_1 one species has *Wolb*, P_2 both species have *Wolb*.

$$P_0 = p_0 P_{even}{}^2 + (1 - p_0) P_{odd}{}^2 =$$

$$p_0\left(\frac{1}{4}\left(1 + e^{-T\mu}\right)^2\right) + (1 - p_0)\left(\frac{1}{4}\left(1 - e^{-T\mu}\right)^2\right) = \frac{1}{2} e^{-T\mu}\left(-1 + 2 p_o + \text{Cosh}[T\mu]\right)$$

$$P_1 = 2 p_0 \left(P_{even} P_{odd}\right) + 2(1 - p_0)\left(P_{even} P_{odd}\right) = \frac{1}{2}\left(1 - e^{-2T\mu}\right)$$

$$P_2 = (1 - p_0) P_{even}{}^2 + p_0 P_{odd}{}^2 =$$

$$(1 - p_0)\left(\frac{1}{4}\left(1 + e^{-T\mu}\right)^2\right) + p_0\left(\frac{1}{4}\left(1 - e^{-T\mu}\right)^2\right) = \frac{1}{2} e^{-T\mu}\left(1 - 2 p_0 + \text{Cosh}[T\mu]\right)$$

The simplification used above is:

In[∘]:= $\text{FullSimplify}\left[(1 - p)\left(\frac{1}{4}\left(1 + e^{-T\mu}\right)^2\right) + (p)\left(\frac{1}{4}\left(1 - e^{-T\mu}\right)^2\right)\right]$

Out[∘]= $\frac{1}{2} e^{-T\mu}\left(1 - 2 p + \text{Cosh}[T\mu]\right)$

Check: these probabilities sum to one as they should:

In[∘]:=
```
Simplify[1/2 e^(-T μ) (-1 + 2 p + Cosh[T μ]) + 1/2 (1 - e^(-2 T μ)) + 1/2 e^(-T μ) (1 - 2 p + Cosh[T μ])]
```

Out[∘]= 1

A likelihood function

We can interpret the probability of a species *Wolbachia* status above as a likelihood of μ, given T_i and an observed infections status):

$L[\mu \mid T_i, WS_i] = p[WdS_i \mid T, \mu]$

The likelihood of μ across taxa is simply a product across all taxa:

$L[\mu] = \prod_i L[\mu \mid T_i, WS_i]]$

Assume that there is an equilibrium fraction of infected taxa mu

In[13]:=
```
likWolb2[{inf_, T_}, μ_, p_] := If[inf == 1 ,
    1/2 - 1/2 e^(-2 T μ),
    If[inf == 0,
     1/2 e^(-T μ) (-1 + 2 p + Cosh[T μ]),
     1/2 e^(-T μ) (1 - 2 p + Cosh[T μ]) ]] // N;
```

Fitting to *Wolbachia* data

In[6]:=
```
(pairTab = Import[
    "/home/klohse/Dropbox/Manuscripts/Eric_Wolbachia_2023/Table_S4_Species_pairs_traits.csv
     ", "CSV"]) // TableForm
```

Out[6]//TableForm=

Genus	Species 1	π sp.1	Gen y-1 sp.1	Species 2
Brenthis	Brenthis daphne	0.0046	1	B. ino
Colias	Colias alfacariensis	0.0243	2-3	C. hyale
Erebia	Erebia ligea			E. euryale
Euchloe	Euchloe ausonia	0.025	2	E. crameri
Fabriciana	Fabriciana adippe			F. niobe
Gonepteryx	Gonepteryx cleopatra	0.0104	1	G. rhamni
Iphiclides	Iphiclides feisthamelii	0.0079	1-3	I. podalirius
Lasiommata	Lasiommata megera	0.0385	2-3	L. petropolitar
Melanargia	Melanargia galathea	0.0152	1	M. lachesis
Pieris	Pieris mannii	0.01	3	P. rapae
Polyommatus	Polyommatus eros	0.0104	1	P. icarus
Pontia	Pontia daplidice	0.0063	3	P. edusa
Pseudophilotes	Pseudophilotes baton	0.008	1-2	P. panoptes
Pyrgus	Pyrgus malvae	0.0164	1-2	P. malvoides
Satyrus	Satyrus actaea	0.0261	1	S. ferula
Spialia	Spialia orbifer	0.0331	2	S. sertorius
Thymelicus	Thymelicus acteon	0.0154	2	T. sylvestris
Zerynthia	Zerynthia cassandra	0.0033	1	Z. polyxena

I am removing *Fabriciana* and *Erebia* given that we have no divergence time estimates for these pairs:

In[4]:=
```
simplStatus[ll_] := If[ll[[1]] == 0 && ll[[2]] == 0, 0, If[ll[[1]] > 0 && ll[[2]] > 0, 2, 1]];
```

In[7]:=
```
divT = #[[10]] & /@ Drop[Delete[Delete[pairTab , 6], 4], 1]
```

Out[7]= {3.03448 × 10^6, 2.74138 × 10^6, 7.17241 × 10^6, 5.44828 × 10^6, 3.51724 × 10^6, 5.44828 × 10^6, 3.91379 × 10^6, 9.05172 × 10^6, 6.58621 × 10^6, 6.91379 × 10^6, 2.94828 × 10^6, 3.31034 × 10^6, 8.5 × 10^6, 5.03448 × 10^6, 1.1569 × 10^7, 4.81034 × 10^6}

In[8]:=
```
statInf = Drop[#, 22] & /@ Drop[Delete[Delete[pairTab , 6], 4], 1]
```

Out[8]= {{0, 0, 0}, {2, 3, 1}, {0, 1, 0}, {2, 0, 0}, {1, 2, 1}, {1, 3, 0}, {4, 2, 1}, {0, 0, 0}, {2, 1, 0}, {0, 1, 0}, {1, 0, 0}, {2, 0, 0}, {0, 1, 0}, {1, 0, 0}, {1, 1, 0}, {0, 0, 0}}

In[9]:=
```
status = simplStatus[#] & /@ statInf
```

Out[9]= {0, 2, 1, 1, 2, 2, 2, 0, 2, 1, 1, 1, 1, 1, 2, 0}

In[10]:=
```
data = {status,  divT} // Thread;
```

I am assuming that the probability that an ancestral species is infected is simply given by the fraction

of species in which *Wolb* has been found, i.e. there is an equilibrium:

In[11]:=
```
prev = Mean[status / 2] // N
```
Out[11]=
```
0.59375
```

In[14]:=
```
muEst =
  FindMaximum[{Total[Table[Log[likWolb2[data[[i]], μ, 1 - prev]], {i, 16}]], 0.0000001 > μ > 0}, {μ}]
```
Out[14]=
```
{-17.2182, {μ → 1.09999 × 10^-7}}
```

The likelihood surface has a single peak:

In[23]:=
```
Plot[Total[Table[Log[likWolb2[data[[i]], μ, 1 - prev]], {i, 16}]], {μ, 0, 0.000001}]
```
Out[23]=

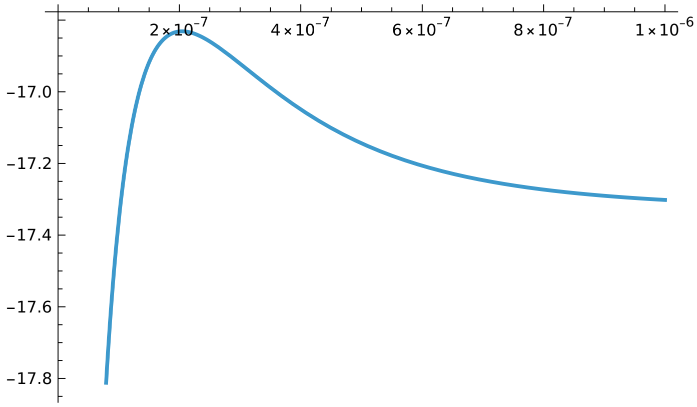

This implies an average time for *Wolbachia* infection status to change from infected to uninfected of 9million years.

In[∘]:=
```
1 / muEst[[2, 1, 2]]
```
Out[∘]=
```
9.09097 × 10^6
```

A simple Poisson model of strain gain and loss

An alternative is to count the number of different strains between sister species and assume that the number of strain gains (or losses) is Poisson distributed.

In[16]:=
```
gainsAndlosses[ll_] := (#[[1]] + #[[2]] - #[[3]]) & /@ ll;
```

In[17]:=
```
numevents = gainsAndlosses[statInf];
data3 = {numevents, divT} // Thread
```
Out[18]=
```
{{0, 3.03448 × 10^6}, {4, 2.74138 × 10^6}, {1, 7.17241 × 10^6}, {2, 5.44828 × 10^6},
 {2, 3.51724 × 10^6}, {4, 5.44828 × 10^6}, {5, 3.91379 × 10^6}, {0, 9.05172 × 10^6},
 {3, 6.58621 × 10^6}, {1, 6.91379 × 10^6}, {1, 2.94828 × 10^6}, {2, 3.31034 × 10^6},
 {1, 8.5 × 10^6}, {1, 5.03448 × 10^6}, {2, 1.1569 × 10^7}, {0, 4.81034 × 10^6}}
```

In[19]:=
```
likWolbPois[{k_, T_}, μ_] := (Exp[-μ 2 * T] * (μ 2 * T)^k) / k!
```

In[20]:=
```
muPoisEst =
  FindMaximum[{Total[Table[Log[likWolbPois[data3[[i]], μ]], {i, 16}]], 0.000004 > μ > 0}, {μ}]
```

Out[20]=

$\{-32.3203, \{\mu \to 1.61111 \times 10^{-7}\}\}$

In[24]:=
```
Plot[Total[Table[Log[likWolbPois[data3[[i]], μ]], {i, 16}]], {μ, 0, 0.000001}]
```

Out[24]=

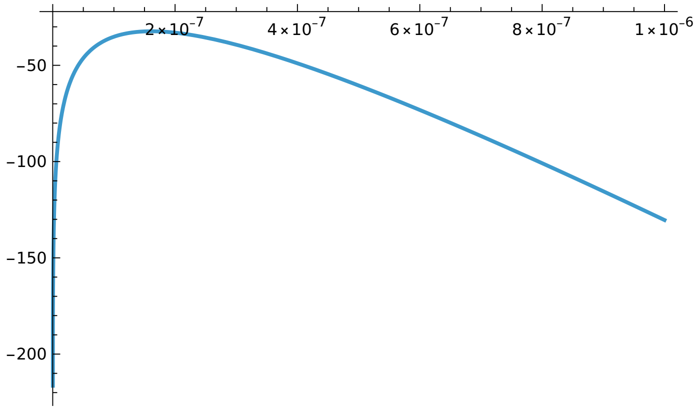

This translates into an turnover rate of 1/6 million generations:

In[21]:=
```
1 / muPoisEst[[2, 1, 2]]
```

Out[21]=

6.20689×10^{6}

This back of the envelope calculation is better than the model above because we assume that each strain by which species in a pair differ must be due to at least one gain or loss.

Check: we can get the same rate estimate by dividing the total amount of host evolution all pairs span which is (twice) the sum of divergence times by the total number of gains and losses:

In[22]:=
```
Total[numevents] / (2 * Total[divT])
```

Out[22]=

1.61111×10^{-7}
