# Supplementary material for: Wolbachia Host Shifts and Widespread Occurrence of Reproductive Manipulation Loci in European Butterflies
Source: Mol Ecol. 2025 Oct 8;34(21):e70125. doi: 10.1111/mec.70125 (PMC12573729; doi:10.1111/mec.70125)
Supplement: Supplementary file 1 — Data S1: mec70125‐sup‐0001‐DataS1.zip. [file MEC-34-e70125-s001.zip › mec70125-sup-0001-DataS1/mec70125-sup-0001-Figures.pdf]

Supplementary Materials for: *Wolbachia* host shifts and widespread occurrence of reproductive manipulation loci in European butterflies

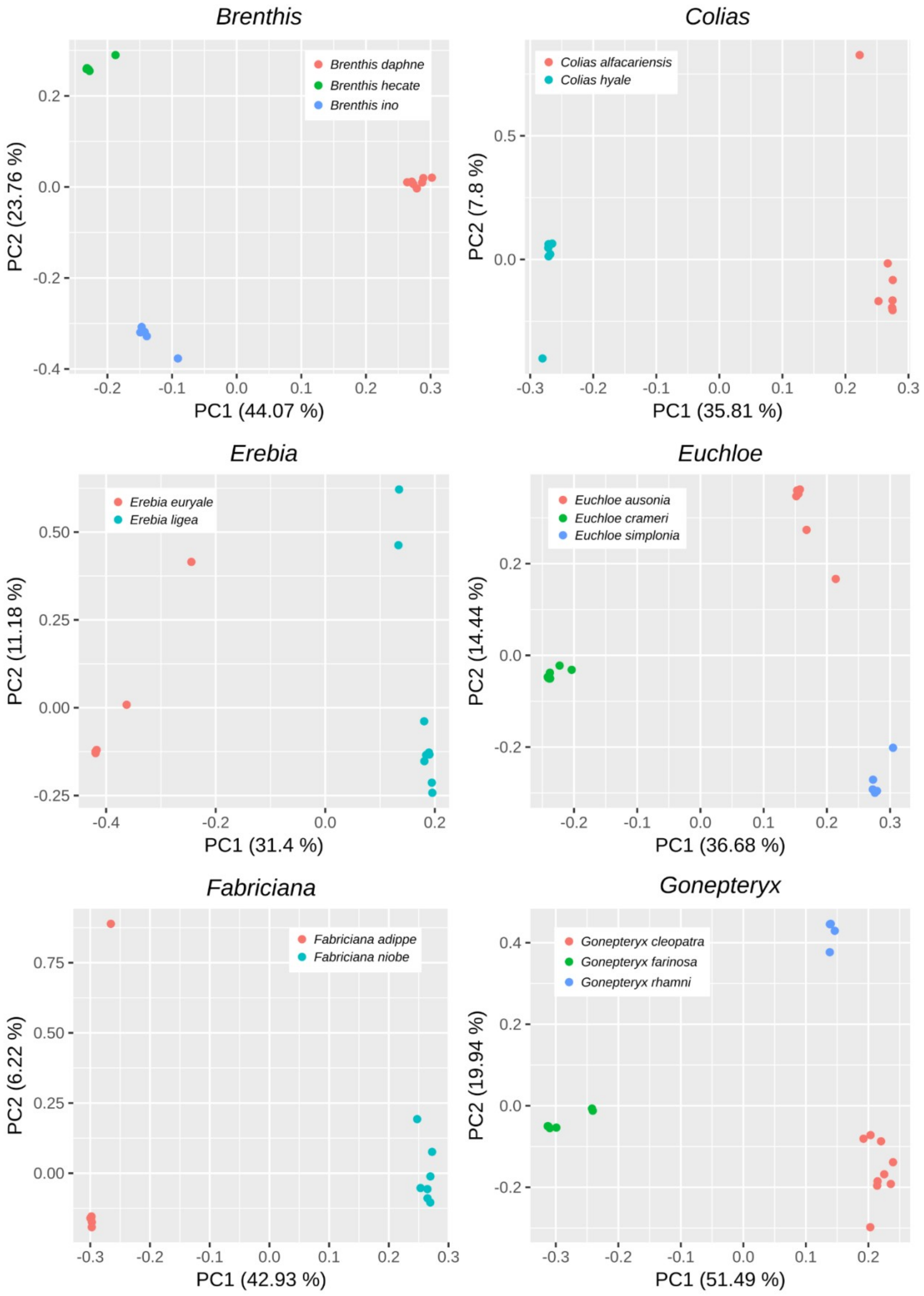

Figure S1. Principal component analysis (PCA) of the genetic variation for each of the studied butterfly genera. The first (x-axis) and second (y-axis) principal components are shown for each genus, with the percent of variance explained by each component in the axis labels.

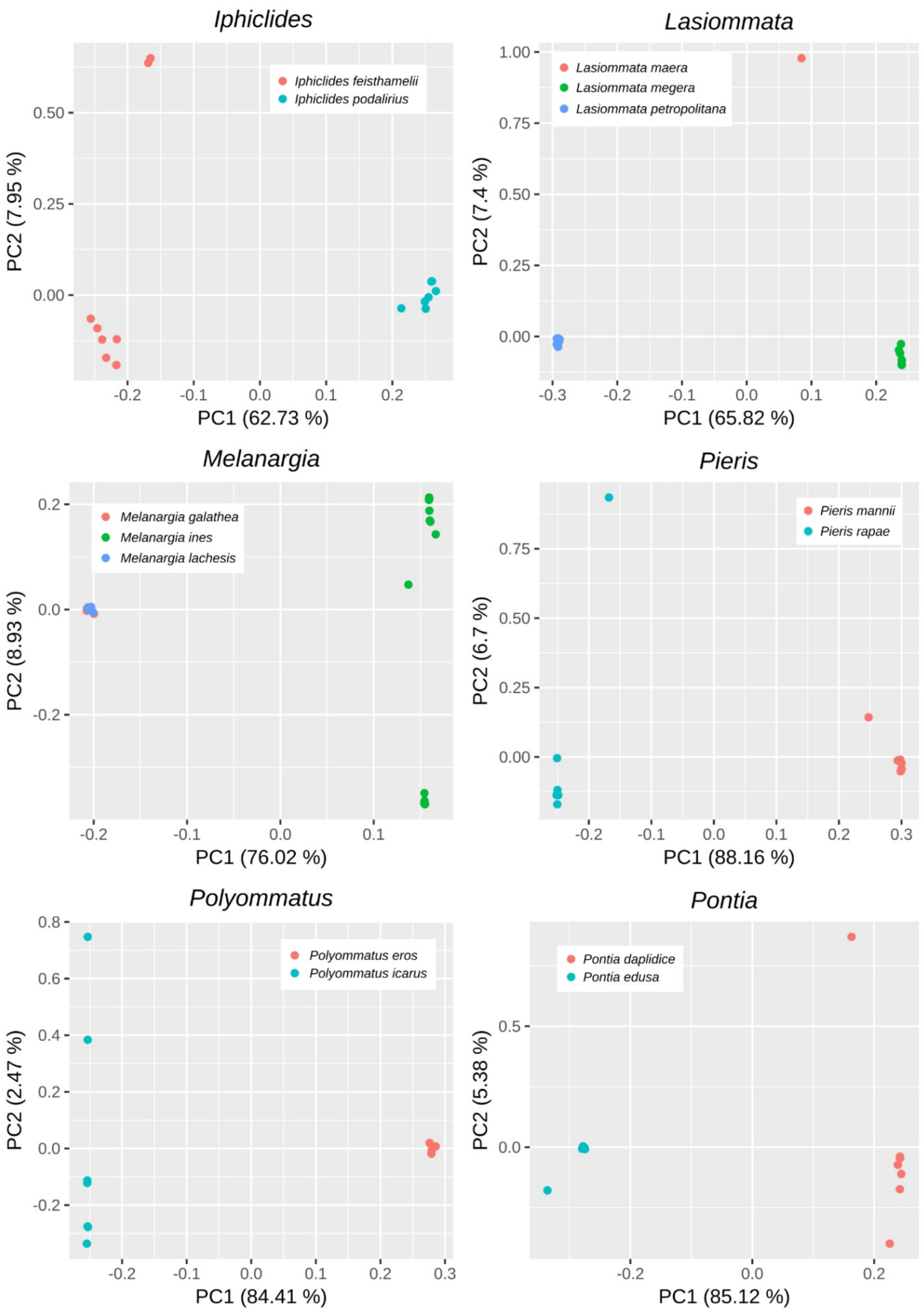

Figure S1 (continued). Principal component analysis (PCA) of the genetic variation for each of the studied butterfly genera. The first (x-axis) and second (y-axis) principal components are shown for each genus, with the percent of variance explained by each component in the axis labels.

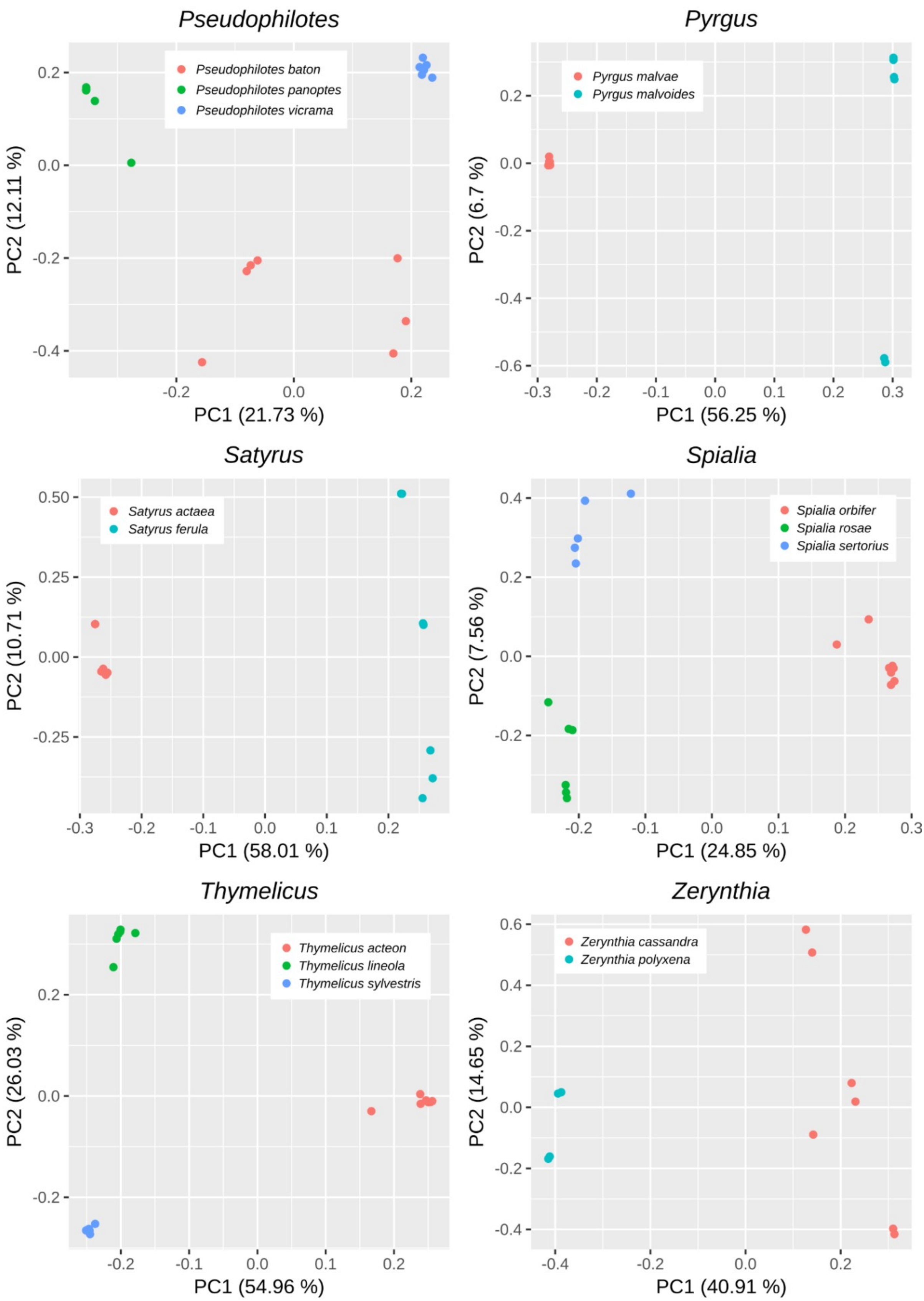

Figure S1 (continued). Principal component analysis (PCA) of the genetic variation for each of the studied butterfly genera. The first (x-axis) and second (y-axis) principal components are shown for each genus, with the percent of variance explained by each component in the axis labels.

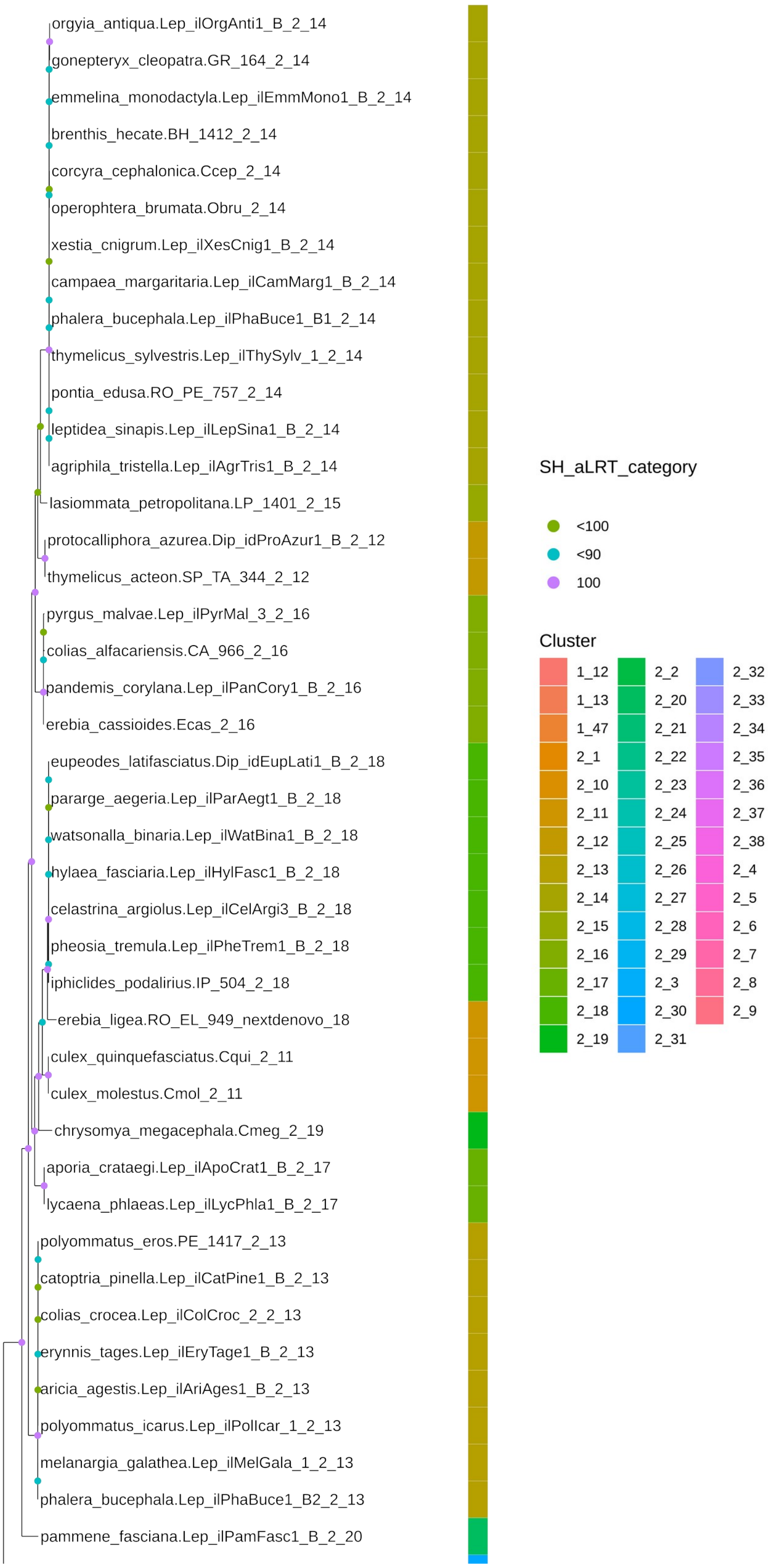

Figure S2. Phylogenetic tree of the supergroup B *Wolbachia* genomes, reconstructed using Maximum Likelihood. Node colors indicate bootstrap support, while the heatmap indicates the clusters formed by dRep based on an average nucleotide identity (ANI) threshold of 99%. Cluster names are also indicated at the end of each tip label.

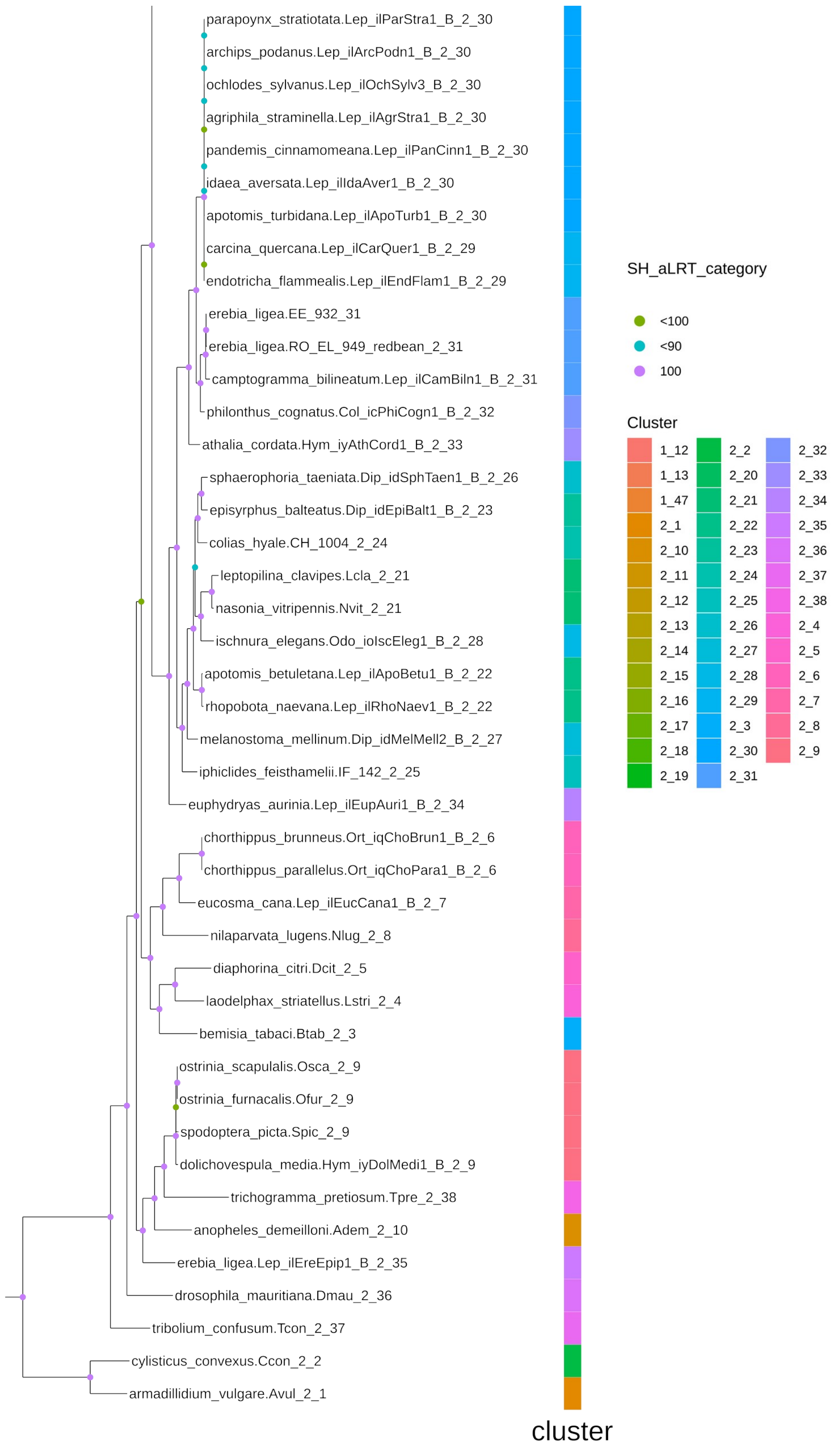

Figure S2 (continued). Phylogenetic tree of the supergroup B *Wolbachia* genomes, reconstructed using Maximum Likelihood. Node colors indicate bootstrap support, while the heatmap indicates the clusters formed by dRep based on an average nucleotide identity (ANI) threshold of 99%. Cluster names are also indicated at the end of each tip label.

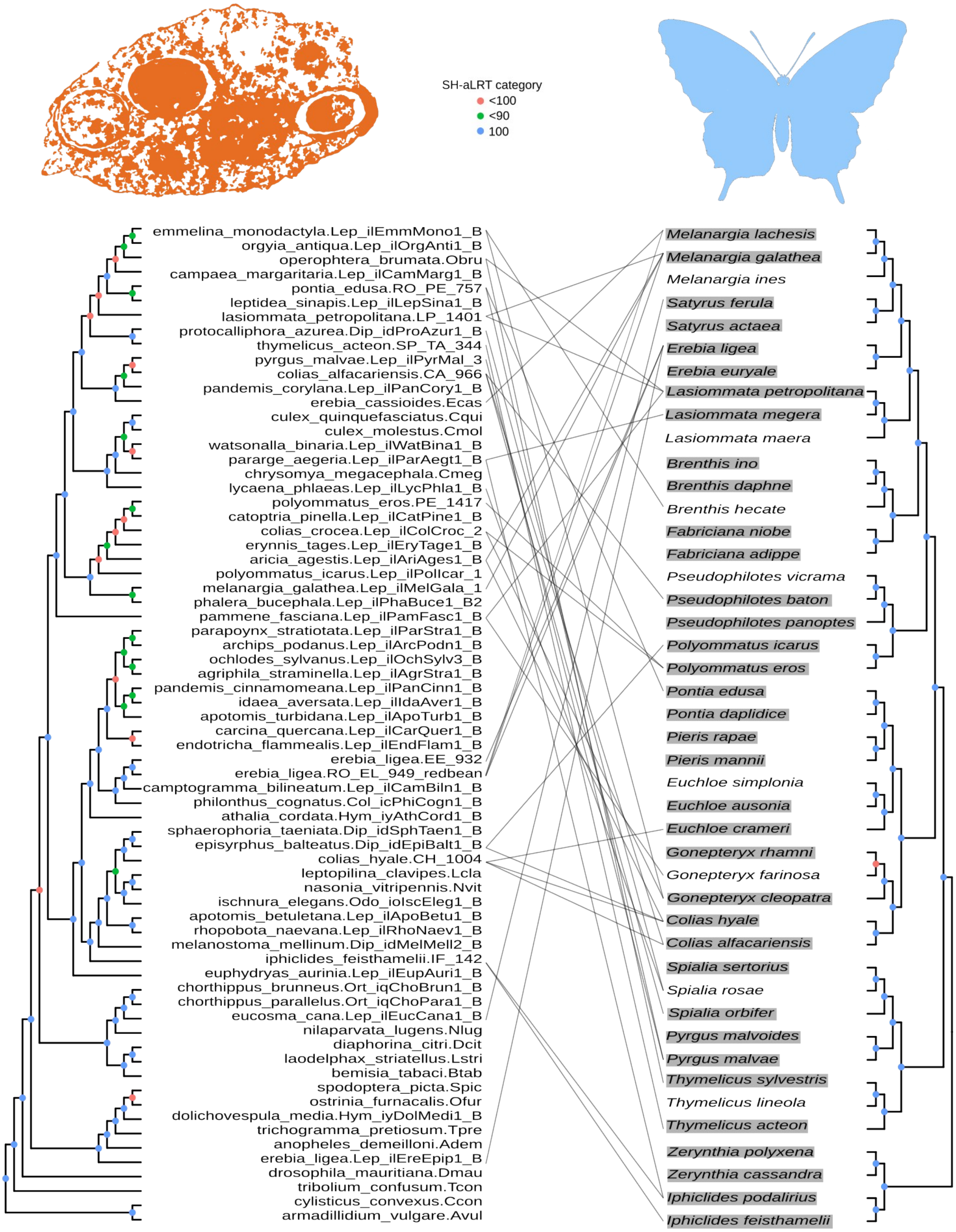

Figure S3. Tanglegram between the *Wolbachia* supergroup B phylogeny (left) and the butterfly phylogeny (right). The *Wolbachia* phylogeny includes only the representative genomes of each of the clusters obtained from dRep. Nodes are coloured according to their bootstrap support (SH-aLRT category) based on 1,000 replicates. The lines connect each infected butterfly species to the *Wolbachia* genomes that are considered to represent the strains found in each butterfly species. Grey boxes on the butterfly phylogeny highlight the sister species pairs.

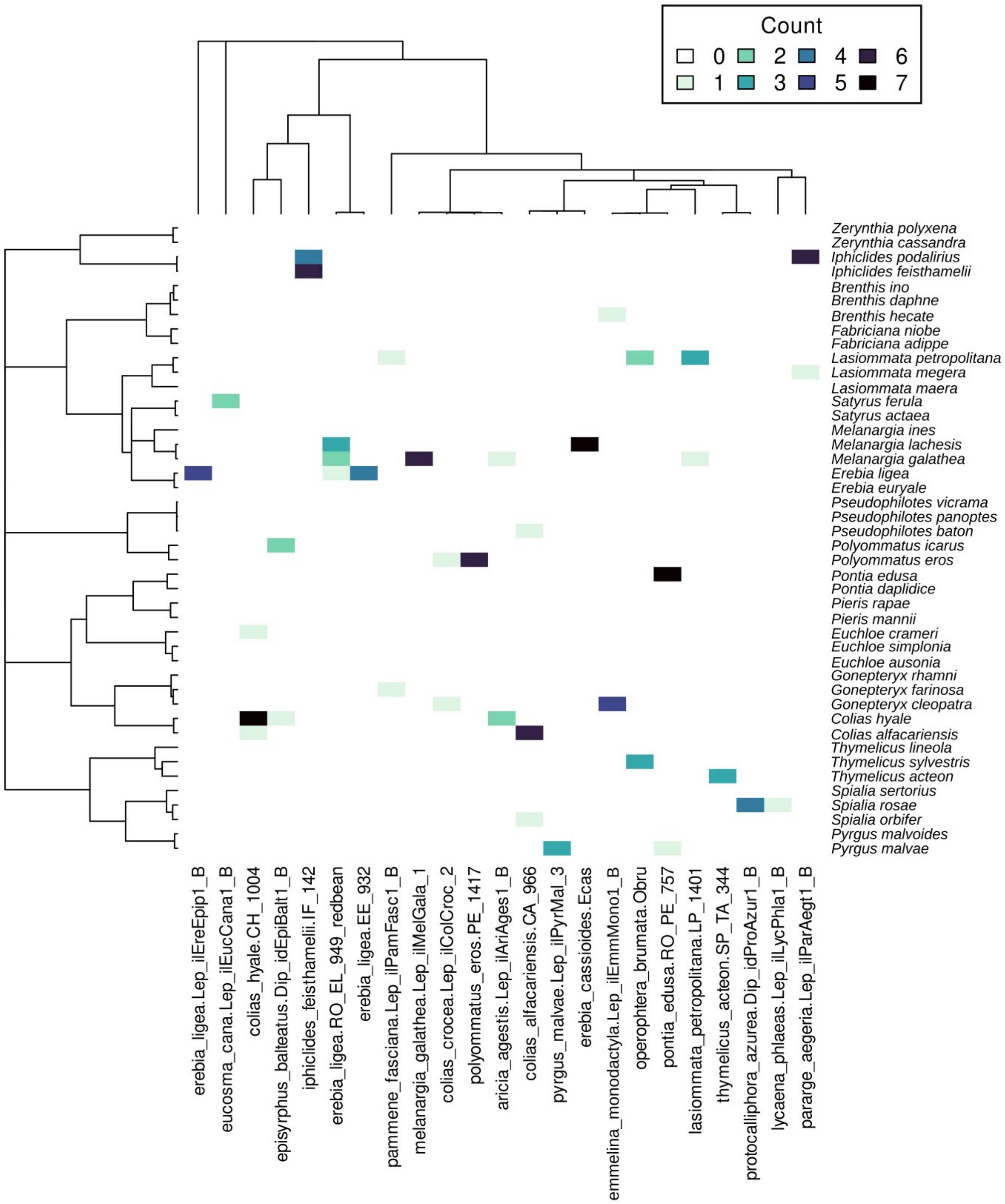

Figure S4. Interaction matrix between *Wolbachia* strains (columns) and butterfly hosts (rows). Each cell in the matrix is colored according to the number of specimens of the butterfly host that harbored the corresponding strain. The strains are ordered according to their phylogenetic relationships, shown at the top of the figure. The butterfly hosts are ordered based on their mitochondrial phylogenetic relationships, shown at the left of the figure.

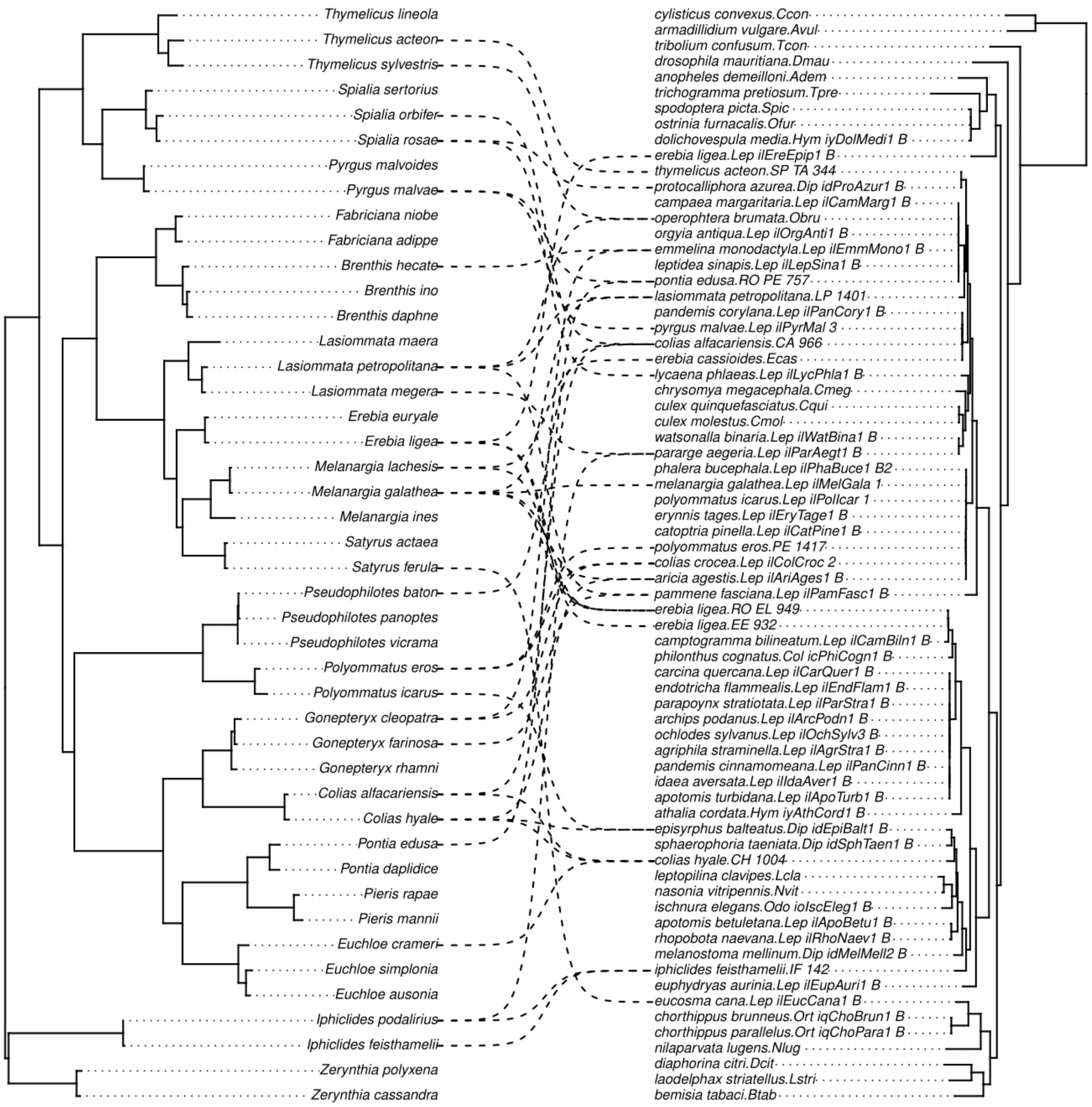

Figure S5. Tanglegram of the *Wolbachia* phylogeny against the mitochondrial butterfly phylogeny.

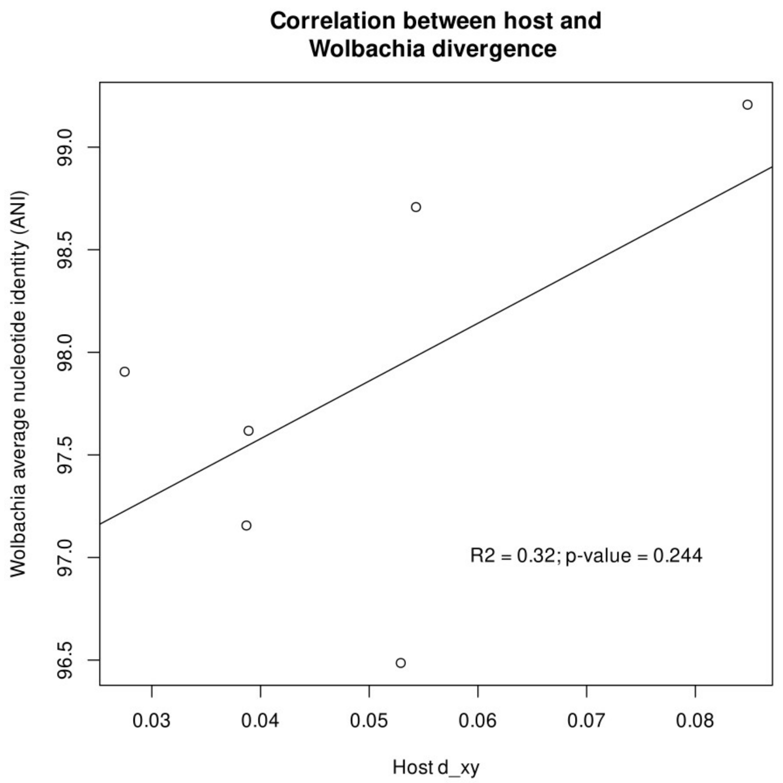

Figure S6. Correlation between host d_{xy} (obtained from Ebdon *et al*., 2021) and *Wolbachia* average nucleotide identity (ANI) computed by FastANI.

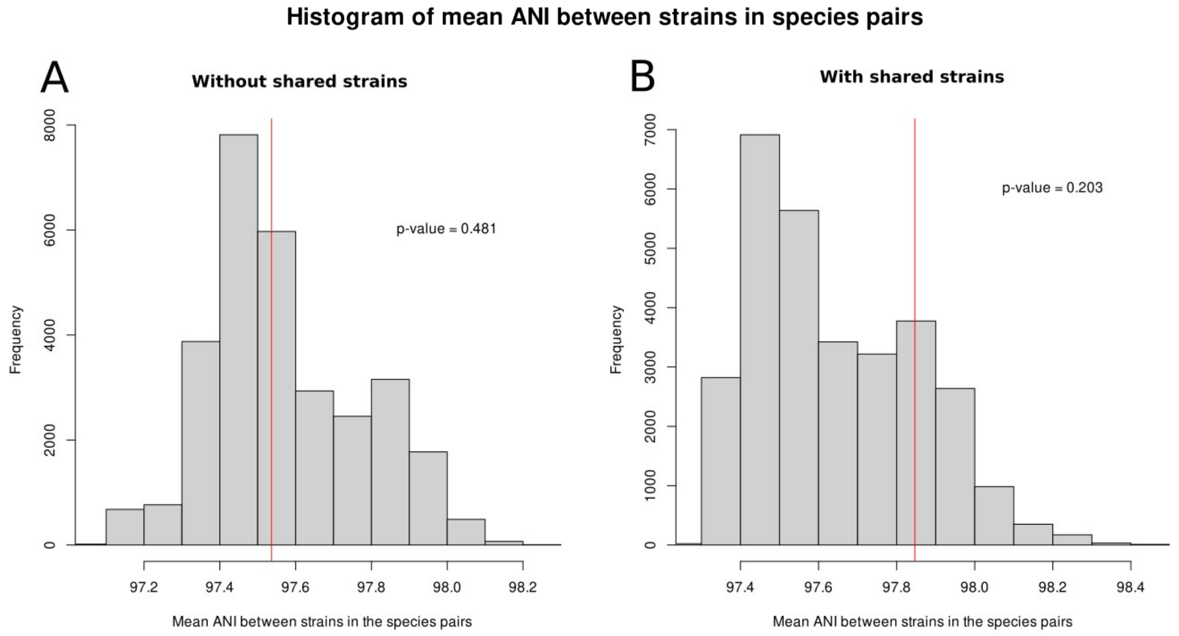

Figure S7. Histograms of the mean average nucleotide identity (ANI) between the strains found in a species pair A) when not considering strains shared by the pair and B) when considering them. The vertical red line indicates the empirical value for the “true” sister species pairs.

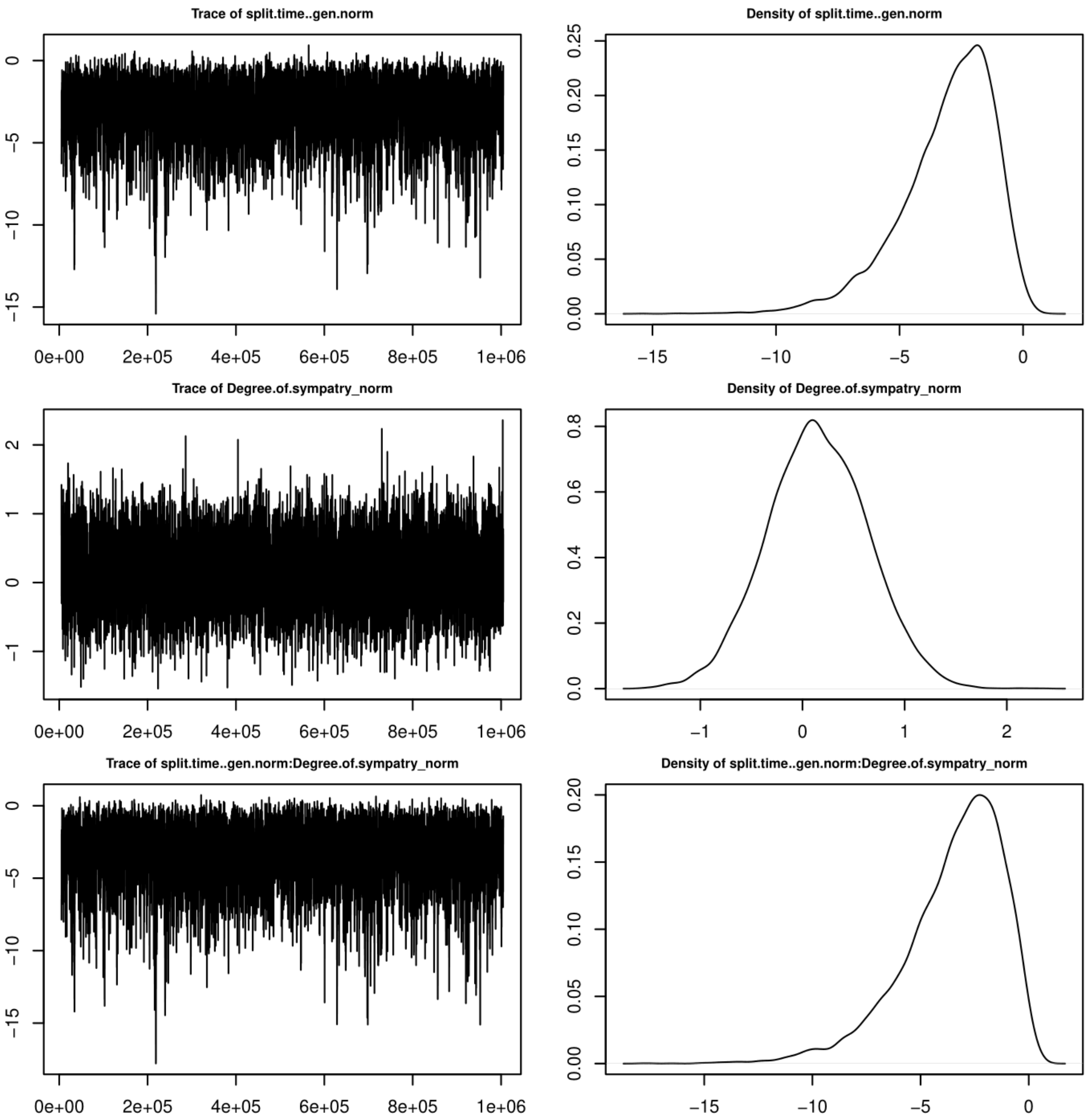

Figure S8: Trace plots (left) and posterior distributions (right) for the coefficients of the MCMCglmm model of strain sharing when split times were measured in number of generations. The trace plots show the value of the model's coefficients for each term (y-axis) throughout the generations of the Markov chain (x-axis). The plots are flat (i.e. the value of the parameter stays around the same area) but move up and down frequently, indicating proper mixing of the chain. The posterior distributions show the values obtained for the coefficients throughout the generations, but in the form of a density plot. Negative values indicate a negative effect of the given term on the probability of sharing strains, and vice versa. For example, the mostly negative values of the posterior distribution of the coefficient for split time indicate that increasing split time decreases the probability of sharing strains.

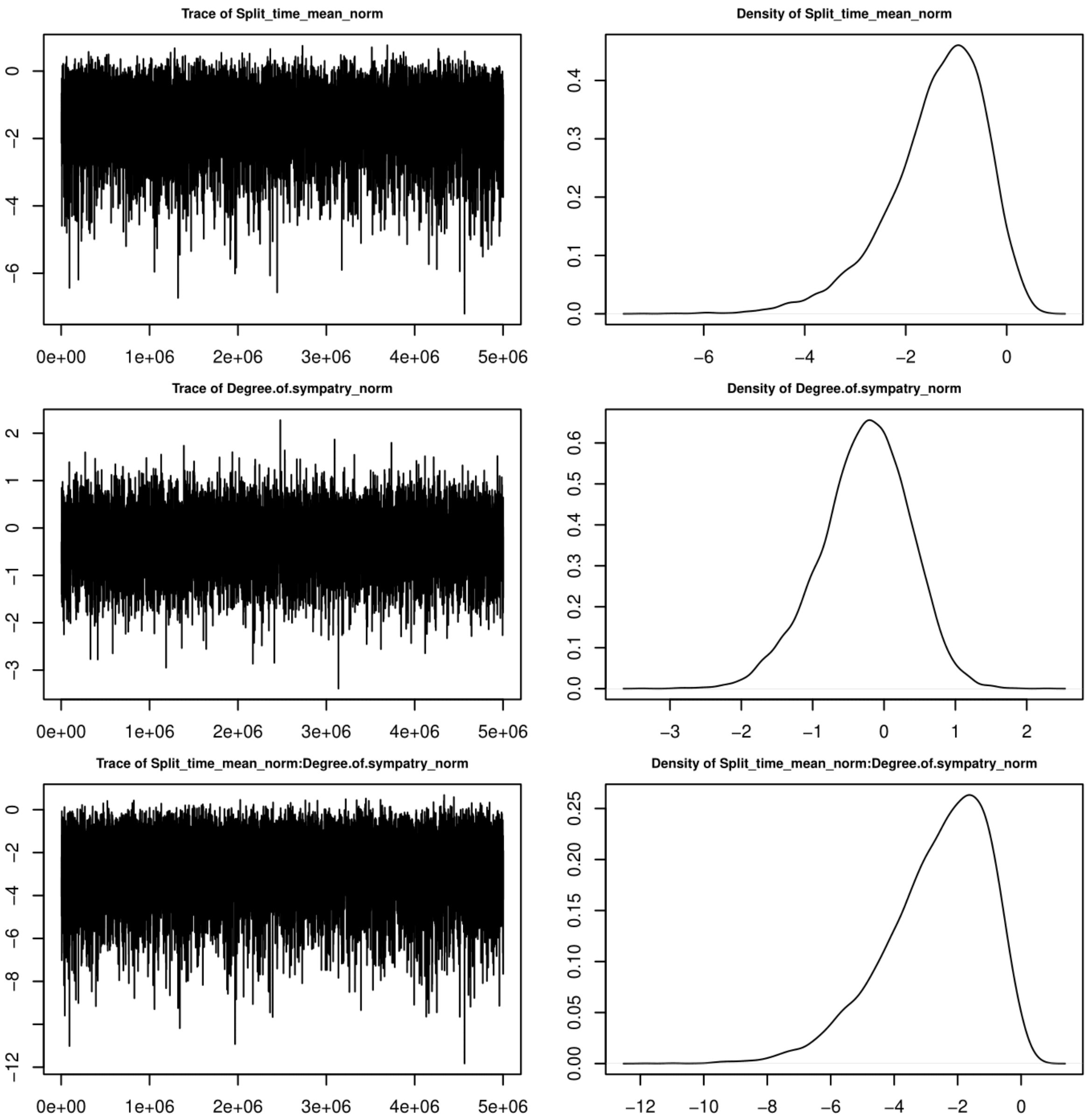

Figure S9: Trace plots (left) and posterior distributions (right) for the coefficients of the MCMCglmm model of strain sharing when split times were measured in million years. The trace plots show the value of the model's coefficients for each term (y-axis) throughout the generations of the Markov chain (x-axis). The plots are flat (i.e. the value of the parameter stays around the same area) but move up and down frequently, indicating proper mixing of the chain. The posterior distributions show the values obtained for the coefficients throughout the generations, but in the form of a density plot. Negative values indicate a negative effect of the given term on the probability of sharing strains, and vice versa. For example, the mostly negative values of the posterior distribution of the coefficient for split time indicate that increasing split time decreases the probability of sharing strains.

Supplementary Tables

Table S1. List of all butterfly specimens analysed, with their *Wolbachia* infection status and metadata. The meaning of the columns is as follows: Sample ID, the code used to identify a particular sample in the dataset; Genus, the taxonomic genus of the sample; Species, the specific epithet of the species to which that sample belongs; Reference_individual, whether that sample corresponds to a reference sample, for which PacBio Sequel libraries were also generated; Clear_absence, whether that sample presented very low depth and breadth of coverage when mapped against *Wolbachia* genomes and thus was confidently considered as not infected; large NUWTs present, whether this sample presented large NUWTs (nuclear *Wolbachia* transfers); Number of *Wolbachia* strains, number of strains detected in the sample; Closest reference *Wolbachia* strains, the name of the *Wolbachia* genome representing the cluster that obtained the highest breadth of coverage in the competitive mapping stage, coinfections are separated with commas; Host nuclear mean coverage (Illumina), the mean coverage obtained when mapping the Illumina library against the butterfly genome of that species or, when not available, the closest genome available; Phenotypic Sex, the sex of the butterfly specimen based on external morphology; Genetic Sex, the sex of the butterfly specimen based on coverage of the sex chromosomes; Date, the date in which the specimen was collected; Collector, the person who collected the specimen; Locality, the name of the locality in which the specimen was collected; Island, the name of the island (when applicable) in which the specimen was collected; State, the name of the state in which the specimen was collected; Country, the name of the country in which the specimen was collected; Continent, the name of the continent in which the specimen was collected; Altitude, the altitude at the point of collection of the specimen; Latitude, the latitude of the coordinates of the point of collection of the specimen; Longitude, the longitude of the coordinates of the point of collection of the specimen.

See Table_S1_Sample_metadata.xlsx

Table S2. Table S2. Quality metrics of the obtained genome assemblies. Host genus, the genus to which the host of the Wolbachia strain belongs; host species, the species to which the host of the Wolbachia strain belongs; ID, the identifier of the specimen that was sequenced; Assembler, the assembly software used to generate this assembly; Polisher, the polishing software used to generate this assembly; N contigs (>1kbp), the number of contigs greater than 1,000 base pairs contained in this assembly; Span (>1kbp), the total length, in base pairs, of the assembly, considering only contigs longer than 1kbp; N50, the scaffold N50; Longest scaffold, the longest scaffold comprised in the assembly; GC%, the precentage of guanine and cytosine of the assembly; BUSCO C, the count of BUSCO genes recovered as complete for this assembly; BUSCO C%, the percentage of BUSCO genes recovered as complete for this assembly over the total of BUSCO genes inthe rickettsiales_odb10 database; BUSCO S, the count of BUSCO genes recovered as single copy for this assembly; BUSCO D, the count of BUSCO genes recovered as duplicated for this assembly; BUSCO F, the count of BUSCO genes recovered as fragmented for this assembly; BUSCO M, the count of BUSCO genes recovered as missing for this assembly; BUSCO M %, the percentage of BUSCO genes missing for this assembly over the total of BUSCO genes inthe rickettsiales_odb10 database; N of CDS, the number of coding sequences annotated in this assembly by Prokka; N hypothetical proteins, the number of coding sequences that were annotated as "hypothetical" in this assembly by Prokka; % CDS with hit on references, the number of coding sequences that were annotated based on putative homologs from reference Wolbachia genomes; proteome span, the total length (in aminoacids) of the annotated CDSs; mean protein length, the mean protein length of the proteins in this assembly annotated by Prokka; N of rRNA, the number of rRNAs annotated by Prokka; N of tRNA, the number of tRNAs annotated by Prokka; Assembled by, the name of the person who assembled this Wolbachia genome; this corresponds to the first author except when the genome was already present in nuclear butterfly genome assemblies; Final, whether this assembly is the final one, used for downstream analyses of this study, or not..

See Table_S2_genome_assemblies.xlsx

Table S3. Quality metrics of the obtained mitochondrial genome assemblies. The seed column only applied to genomes assembled with NOVOplasty, which requires a sequence to use as seed for starting the assembly. The column “Input assembly” only applies to those genomes recovered from a previously assembled dataset with MitoHifi -c option. The meaning of the columns is as follows: Specimen, the ID code of the specimen used to assemble the mitogenome; Species, the species to which the mitogenome being assembled belongs; Assembly_method, the tool used to assemble the mitogenome (in the case of MitoHifi, it was used to extract a mitogenome from a preexisting genome assembly); Annotation_method, the tool used to annotate the mitochondrial genes, including whether or not manual curation of the annotation was conducted; Seed, the seed sequence used to start the assembly (only applicable to NOVOPlasty); Input_assembly, the name of the input assembly form which mitochondrial sequences were extracted when using MitoHifi; Contig_number, the final number of contigs conforming the mitogenome; Span_bp, the length (in base pairs) of the assembled mitogenome; Gene_count, the number of genes annotated in the mitogenome; Missing_genes (excluding tRNAs), a list of protein coding genes or rRNAs that were missing in the annotation; Internal stop codons, a list of genes that contained internal stop codons as currently annotated (these were either excluded, or trimmed after alignment if the region containing stop codons was due to an overextended sequence in the annotation); Duplicated PCGs; whether any of the protein coding genes were duplicated in the mitogenome; GC%, the percentage of guanine and cytosine in the mitogenome; Comments, any additional comments on the assembly and annotation process.

See Table_S3_butterfly_mitogenomes_assembly.xlsx

Table S4. inStrain analysis of the competitive mapping of the Illumina libraries against the *Wolbachia* genomes. For each genus, there is one sheet containing the results when mapping against the dereplicated set of *Wolbachia* reference genomes. Each sheet contains results for all the samples of that genus separated by an empty row; the columns indicate the reference genome in question, the average depth of coverage it obtained, the observed breadth of coverage (number of bases of that genome covered by at least one read), the expected breadth (theoretical expectation of breadth based on the coverage if that were the genome from which the reads were generated), and the difference between expected and observed breadth (E-O).

See Table_S4_inStrain.xlsx

Table S5. Results of the progressive competitive mapping to determine the number of strains in each of the infected samples. For each genus, samples were mapped first against the top genome obtaining the highest breadth of coverage in the initial competitive mapping (Table S5) and then. If the number of variants per kilobase was greater than 10, they were mapped competitively against the top two genomes with highest breadth. This process was repeated for each sample until the number of variants per kilobase was lower than 10. The names of each sheet indicate the step of this mapping process and the butterfly genus for which this is done. The meaning of the columns is as follows: sample, the identifier of the sample being mapped; length, the length of the reference genome (in base pairs) against which the library is being mapped; het_sites, the number of variants called across that genome; ANI, the average nucleotide identity corresponding to that number of variants; het_sites_per_kb, the number of variants per kilobase of that genome; putative_strain_number, the putative number of strains based on the number of variants per kilobase.

See Table_S5_het_sites.xlsx

Table S6. Genes determined to be involved in cytoplasmic incompatibility and male-killing in different *Wolbachia* strains, used as reference for annotation of homologs in the newly assembled *Wolbachia* genomes.

Phenotype	Gene name	GenBank ID	*Wolbachia* strain	Host species	*Wolbachia* genome ID	Reference
Cytoplasmic incompatibility	cinA	WP_007302980	wPip	*Culex quinquefasciatus*	GCF_000073005	Beckmann et al. (2017)
	cinB	WP_007302979				
	cidA	WP_012481787				
	cidB	WP_012481788				
Male killing	wmk	AAS14326	wMel	*Drosophila melanosgater*	AE017196	Perlmutter et al. (2019)

Table S7. Arguments used to run the different programs involved in the study.

**Program**	**Version**	**Function**	**Example**
FASTP	0.23.2	Read trimming	fastp -i {reads forward} -I {reads reverse} -o {trimmed reads forwards} -O {trimmed reads reverse} --cut by quality5 --cut by quality3 --cut window size 4 --cut mean quality 20 --html {sample name}.html --thread 16
BLASTn	2.13.0+	Similarity search of sequences	blastn -query {ref_genome} -out {outdir} -num_threads 20 -max_target_seqs 10 -max_hsps 1 -db {nt_database} -evalue 1e-25 -outfmt '6 qseqid staxids bitscore std'
minimap2	2.22-r1101	Align PacBio reads	minimap2 -t 20 -ax map-pb {ref_genome} {pacbio_reads}
		Align Illumina paired-end reads (interleaved)	minimap2 -t 20 -ax sr {ref_genome} {sr_reads1}
BlobTools	1.1.1	Create database	blobtools create -i {ref_genome} -t {blast_results} -b {pacbio_bamfile} -b {illumina_bamfile} -o {out_folder}
		Create taxonomic assignment files	blobtools view -i {blobDB} -o {out_prefix} -r all --hits
		Make BlobPlots	blobtools plot -i {blobDB} -o {out_prefix} -r order
		Partition Illumina paired-end reads	blobtools bamfilter -b {illumina_bamfile} -i {contig_IDs} -o {outfile}

Flye	2.9.1-b1780	Long-read metagenome assembly	flye --pacbio-raw {pacbio_reads} --out-dir {out_dir} -t 40 -i 1 --meta
NextDenovo (config file)	2.4.0	Long-read genome assembly	[General]<br>job_type = local<br>job_prefix = {out_prefix}<br>task = all # 'all', 'correct', 'assemble'<br>rewrite = yes # yes/no<br>deltmp = yes<br>rerun = 0<br>parallel_jobs = 5<br>input_type = raw<br>read_type = clr<br>input_fofn = {input_files_list.fofn}<br>workdir = {work_dir}<br><br>[correct_option]<br>read_cutoff = 1k<br>genome_size = 1500000<br>pa_correction = 5<br>sort_options = -m 40g -t 20 -k 80<br>minimap2_options_raw = -t 12<br>correction_options = -p 13<br>seed_cutoff = 10000<br><br>[assemble_option]<br>minimap2_options_cns = -t 12<br>nextgraph_options = -a 1
HAPO-G	1.3.4	Assembly polishing	hapog --genome {assembly} --pe1 {illumina_reads1} --pe2 {illumina_reads2} -u --output {out_file} --threads 20
Pilon	1.24	Assembly polishing	java -Xmx20G -jar ~/pilon-1.24.jar --genome {genome_assembly} --changes --vcf --tracks --fix all,circles --iupac --frags {bam_file_illumina_against_assembly} --output {out_prefix} --outdir {out_dir}
BUSCO	5.4.2	Evaluate genome quality	busco -m genome -i {genome_assembly} -o {out_dir} -l {busco_database} -c 30
Prokka	1.14.6	Genome annotation	prokka -force --outdir {out_dir} --prefix {out_prefix} --cdsrnaolap --addgenes --addmrna --gcode 11 --kingdom bacteria --genus Wolbachia --cpus 20 --mincontiglen 1000 --proteins {reference_proteomes} {genome_assembly}
OrthoFinder	2.5.4	Find single-copy orthologs (SCOs)	orthofinder -f {proteomes_folder} -n wolbachia -p {out_folder} -a 60 -t 60
KinFin	1.1	Recover SCOs with a given missing data	kinfin -g OrthoGroups.txt -c config.txt -s SequenceIDs.txt --target_count 1 --target_fraction 0.95 --min 0 --max 1

<table>
<tr><td></td><td></td><td>percent</td><td></td></tr>
<tr><td>MAFFT</td><td>7.508</td><td>Align SCOs</td><td>mafft --thread 10 --genafpair --maxiterate 1000 {fasta} > {out_file}</td></tr>
<tr><td>FASconCAT-G</td><td>1.05.1</td><td>Build supermatrix</td><td>~/FASconCAT-G/FASconCAT-G_v1.05.1.pl -s -l -p -p -j</td></tr>
<tr><td>SuperCRUNCH</td><td>1.3.2</td><td>Build supermatrix</td><td>python ~/SuperCRUNCH/supercrunch-scripts/Concatenation.py -i {folder_with_alignments} -o {out_folder} --informat fasta --outformat phylip -s dash</td></tr>
<tr><td>BEDtools</td><td>2.30.0</td><td>Generate BED file of extended BUSCO regions</td><td>grep -v "^#" {busco_all_tsv}| awk '$2=="Complete"' | cut -f3,4,5 | bedtools slop -i - -g {genome_file} -b 1000 | bedtools sort -i - -g {genome_file} | bedtools merge -i - > {out_bedfile}</td></tr>
<tr><td>SAMtools</td><td>1.6</td><td>Subset BAM file based on BED file and quality</td><td>samtools view -q 20 -bL {bed_file} {bam_file} > {output_bam} && samtools index {output_bam}</td></tr>
<tr><td rowspan="3">BCFtools</td><td rowspan="3">1.17</td><td>Call variants</td><td>bcftools mpileup --threads 12 -f {genome} {subsetted_bam}| bcftools call --threads 12 -mv -Oz -o {gzipped_vcf}</td></tr>
<tr><td>Normalise and filter variants</td><td>bcftools norm -f {genome} {gzipped_vcf} -Oz | bcftools view - -e 'QUAL<20 | DP<8' -Oz -o {gzipped_filtered_vcf}</td></tr>
<tr><td>Call consensus sequence with IUPAC codes</td><td>cat {genome} | bcftools consensus --haplotype I {gzipped_filtered_vcf} > {consensus_seq}</td></tr>
<tr><td>IQTree</td><td>2.2.0.3</td><td>Build phylogeny</td><td>iqtree -s {supermatrix} -p {partitions_file} -m PROTGAMMAGTR -bb 1000 -bnni -alrt 1000 -nt 30 -safe -pre {out_prefix}</td></tr>
<tr><td>inStrain</td><td>1.6.4</td><td>Evaluate competitive mapping</td><td>inStrain profile {bamfile} {concatenated_genomes} --stb {contigs_to_genomes_map} -o {out_folder} -p 30 --database_mode</td></tr>
<tr><td>dRep</td><td>3.4.3</td><td>Dereplicate Wolbachia genome set</td><td>dRep dereplicate {out_folder} -g {genomes} --S_ani 0.99 --S_algorithm fastANI</td></tr>
<tr><td>FastANI</td><td>1.33</td><td>Compute average nucleotide identity (ANI)</td><td>fastANI --rl reference_genomes.txt --ql query_genomes.txt -o {out_file} --matrix --visualize -t 30</td></tr>
</table>

Table S8. Infection status for all the butterfly species analyzed in this study. Genus, species genus; Species; species specific epithet; Total samples, total number of samples screened for that species; *Wolbachia* presence, binary variable indicating if *Wolbachia* was detected on a given species; Infected samples, number of samples with *Wolbachia*; *Wolbachia* prevalence, proportion of samples infected with *Wolbachia*; Samples with NUWTS, number of samples with NUWTs detected on a given species; NUWT prevalence, proportion of samples in which NUWTs were detected; Number of strains, number of strains detected in a given species; *Wolbachia* in literature, whether a given species is described as infected, uninfected, or is not found in the literature; Reference individual, code of the genomic reference individual of that species; Supergroup (ref. ind.), *Wolbachia* supergroup of the infection in the genomic reference individual of that species; NCBI TaxID, the taxonomic ID number of that species; Supergroup (literature), the supergroup to which the *Wolbachia* infection in the literature belongs; Strain, strain classification from the literature based on MLST and wsp markers; Prevalence in literature, the prevalence of *Wolbachia* in that species found in the literature; Reference, bibliographic reference of the study that reports the infection status of that species; Geographic region, region of origin of the screened specimens in the literature; Closest RefSeq hit, *Wolbachia* RefSeq genome that is the closest match to the one in the genomic reference individuals; DToL, whether a *Wolbachia* genome used in this study was obtained from the Darwin Tree of Life project; Comments, additional comments on the infection status.

See Table_S8_Wolbachia_presence_species_level.xlsx

Table S9. Comparison of the infection status across sister pairs of butterfly species, being infection-conservative. Genus, species genus; Species 1; first species of the pair; π sp.1, nucleotidic diversity of the first species; Gen y^{-1} sp.1; generations per year of the first species; Species 2, second species in the pair; π sp.2, nucleotidic diversity of the second species; Gen y^{-1} sp.2; generations per year of the second species; d_xy, mean genetic divergence between the species in the pair; d_a, net genetic divergence between the species in the pair; split time (gen), split time in number of generations; Split time (MYA), split time in million years ago; F_st, fixation index; Degree of sympatry, proportion of range overlap between the two species; Contact zone, whether the species in the pair have a contact zone; Known to hybridize, whether the two species in the pair are known to produce hybrids; None infected; whether none of the species in the pair are infected with *Wolbachia*; One infected, whether one species in the pair is infected and the other is not; Both infected, whether both species in the pair are infected; Presence of shared strains in the pair; whether the species pair has at least one strain in common; Presence of specific strains within the pair; whether there are strains in at least one species of the pair that are absent in the other species of the pair; Presence of species-specific strains; whether at least one species in the pair has at least one strain that was not detected in any other species (either in the pair or outside); Presence of strains shared outside the pair (e.g. with other genera), whether at least one species in the pair has some strain that is also found in another species not from the pair; strain_num_sp1, number of strains in the first species of the pair; strain_num_sp2, number of species in the second species of the pair; num_shared_strains, number of strains that are found in both species of the pair.

See Table_S9_Species_pairs_traits.xlsx

Table S10. Summary statistics for the MCMCglmm model of strain sharing, with split times measured in number of generations (top rows) and in million years (bottom row). Posterior mean indicates the mean of the posterior distribution obtained from the Markov Chain Monte Carlo (MCMC) process; lower and upper 95% CI indicate the 95% credibility interval of the distribution; effective sample size indicates the effective sample size of the markov chain after thinning and accounting for non-independence of the iterations; pMCMC indicates the p-value based on the values of the posterior distribution.

Split time measure	Term	Posterior mean	lower 95% CI	upper 95% CI	Effective sample size	pMCMC
Generations	Split time	-3.070	-6.936	-0.088	2272	0.014*
	Degree of sympatry	0.146	-0.839	1.080	9454	0.766
	Split time : degree of sympatry	-3.478	-7.957	0.270	2182	0.021*
Million years ago	Split time	-1.385	-3.424	0.201	10000	0.074
	Degree of sympatry	-0.273	-1.544	0.909	9584	0.692
	Split time : degree of sympatry	-2.644	-5.858	0.087	10000	0.021*

Table S11. Split times estimated for each possible pair of *Wolbachia* lines compared across congeneric species in the dataset. Species1, species hosting the first *Wolbachia* line being compared; Strain1, strain to which the first *Wolbachia* line being compared was assigned; Species2, species hosting the first *Wolbachia* line being compared; Strain2, strain to which the second *Wolbachia* line being compared was assigned; total_cases, total number of comparisons involving occurrences of Strain1 and Strain2 in different specimens of Species1 and Species2; min_host_age; the lower bound of the split time of the host species, based on nuclear data; max_host_age, the upper bound of the split time of the host species, based on nuclear data; min_wolbachia_age, the lower bound of the split time of the *Wolbachia* strains; min_wolbachia_age, the lower bound of the split time of the *Wolbachia* strains; max_wolbachia_age, the upper bound of the split time of the *Wolbachia* strains; max_mito_split_time, the upper bound of the split time of the host species, based on mitochondrial data; min_mito_split_time, the lower bound of the split time of the host species, based on mitochondrial data; host_comp, whether this comparison is within a single host species (intraspecific) or across strains found in the sister species (interspecific); wolb_comp, whether the comparison involves different occurrences of the same strain (intrastrain) or different strains (interstrain); genus, the genus of the host species; interpretation, the interpretation given to the comparison of nuclear, mitochondrial, and *Wolbachia* split times (HT: horizontal transfer; IT: introgressive transfer).

See Table_S11_wolbachia_split_time_comparisons.xlsx

Table S12. List of all significant hits of the newly assembled *Wolbachia* genomes against the reference sequences for the cytoplasmic incompatibility and male-killing loci. Genome, the genome to which the hit belongs to; contig, the contig of the genome to which the hit belongs to; homolog, name of the CI or MK gene which generated the hit; start_position, position where the hit starts in the contig; end_position, position where the hit starts in the contig; overlapping_protein, identifier(s) of the protein(s) predicted by Prokka that overlap with the hit; strand, whether the hit is on the forward (+) or reverse (-) strand; same_frame, whether the hit and the overlapping predicted protein are in the same reading frame; e-value, e-value obtained by the hit in the initial tblastn search; comments, additional details on the hits. Consecutive hits of a cifA and a cifB are marked in blue.

See Table_S12_CI_MK_candidates.xlsx
